# Amino Acid Substitutomics: Profiling Amino Acid Substitutions at the Proteomic Scale Unveils Biological Implications and Escape Hypotheses in Cancer

**DOI:** 10.1021/acs.jproteome.6c00455

**Published:** 2026-08-27

**Authors:** Peize Zhao, Shengzhi Lai, Shuaijian Dai, Chen Zhou, Ning Li, Weichuan Yu

**Affiliations:** † Individualized Interdisciplinary Program, The Hong Kong University of Science and Technology, Hong Kong 999077, China; ‡ Department of Electronic and Computer Engineering, The Hong Kong University of Science and Technology, Hong Kong 999077, China; § Division of Life Science, The Hong Kong University of Science and Technology, Hong Kong 999077, China; ∥ Department of Biomedical Engineering, School of Basic Medical Sciences, Central South University, Changsha, Hunan Province 410013, China

**Keywords:** cancer proteome, cancer escape, amino acid substitutomics, amino acid substitutome

## Abstract

Amino acid (AA) substitutions are pivotal modulators of cellular signaling and homeostasis. Current studies of AA substitutions rely mainly on genomic and transcriptomic evidence, while proteome-level characterization remains underexplored, even though such substitutions can arise beyond DNA and RNA mutations. Here we address this gap with PIPI-C, an open-search mass spectrometry tool, and establish an integrated AA substitutomics pipeline to profile AA substitutions across five cancer cohorts and dissect the regulatory roles of substitution-bearing proteins. By cross-referencing 13,399 identified AA substitutions with matched whole-exome sequencing data, we observed no overlap with patient somatic mutations. Specifically, 61% of these substitutions required two or more base alterations and could not be derived from single-nucleotide variants, rendering them exclusively detectable at the proteomic level. The remaining 39% of substitutions were compatible with single-base variations. PIPI-C captured these candidate substitutions at the proteome scale, consistent with a translational-stage origin as reported for alternate RNA decoding in mammals. We further identified notable substitutions, including F43S and E91D in hemoglobin subunit beta, P584T in filamin A, and A175N in fructose-bisphosphate aldolase B, and our pipeline provides a framework for generating hypotheses about AA substitutions in cancer escape.

## Graphical abstract

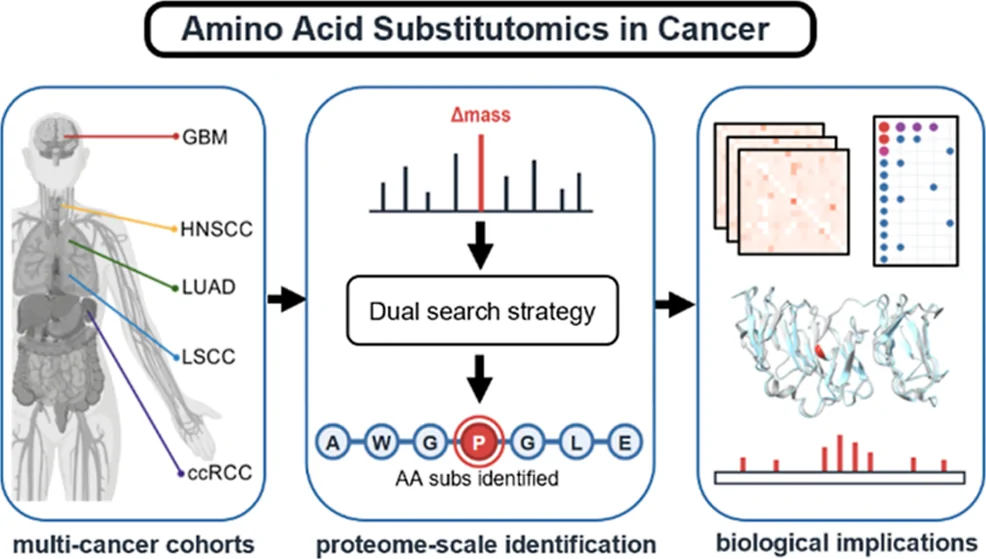

## Introduction

Cancer is driven by the breakdown of the regulatory networks that govern normal growth and homeostasis.^1^ As the primary executors of cellular processes, proteins are central to tumorigenesis, where aberrant expression, mutational damage, or functional dysregulation disrupts division, survival, and apoptosis.^2,3^ Yet proteome-level alterations—particularly amino acid (AA) substitutions—remain incompletely defined, despite their direct impact on protein structure and function.

AA substitutions disrupt native protein structure and function, with a subset conferring selective growth advantages and reinforcing malignant hallmarks.^4–6^ They arise from three biologically distinct sources—DNA mutations, mRNA mutations, and alternate translation—the last of which recent proteome-scale evidence indicates can yield stable, abundant substituted proteins.^7^ The relative contribution of each source has been difficult to quantify; here, cross-referencing against matched somatic mutation data shows that none coincide with a patient somatic mutation, that 61% require two or more base changes and are thus identifiable only by proteome-scale profiling, and that the remaining 39% are single-nucleotide-accessible, for which an uncalled germline or other low-frequency DNA/RNA variant cannot be excluded (Figure 1A). Prior work has been driven largely by multiomics integration: genomics links many AA substitutions to deleterious driver mutations enriched at protein-interaction hubs,^8,9^ transcriptomics adds expression signatures and RNA-editing events,^10,11^ and proteomics has begun to bridge genotype and phenotype—tryptophan-to-phenylalanine (W > F) substitutions modulate immune surveillance^12^ and arginine-to-cysteine (R > C) substitutions are enriched in KEAP1 mutant lung cancers.^13^ However, these approaches remain largely restricted to substitutions of genetic origin.

**Figure 1:**
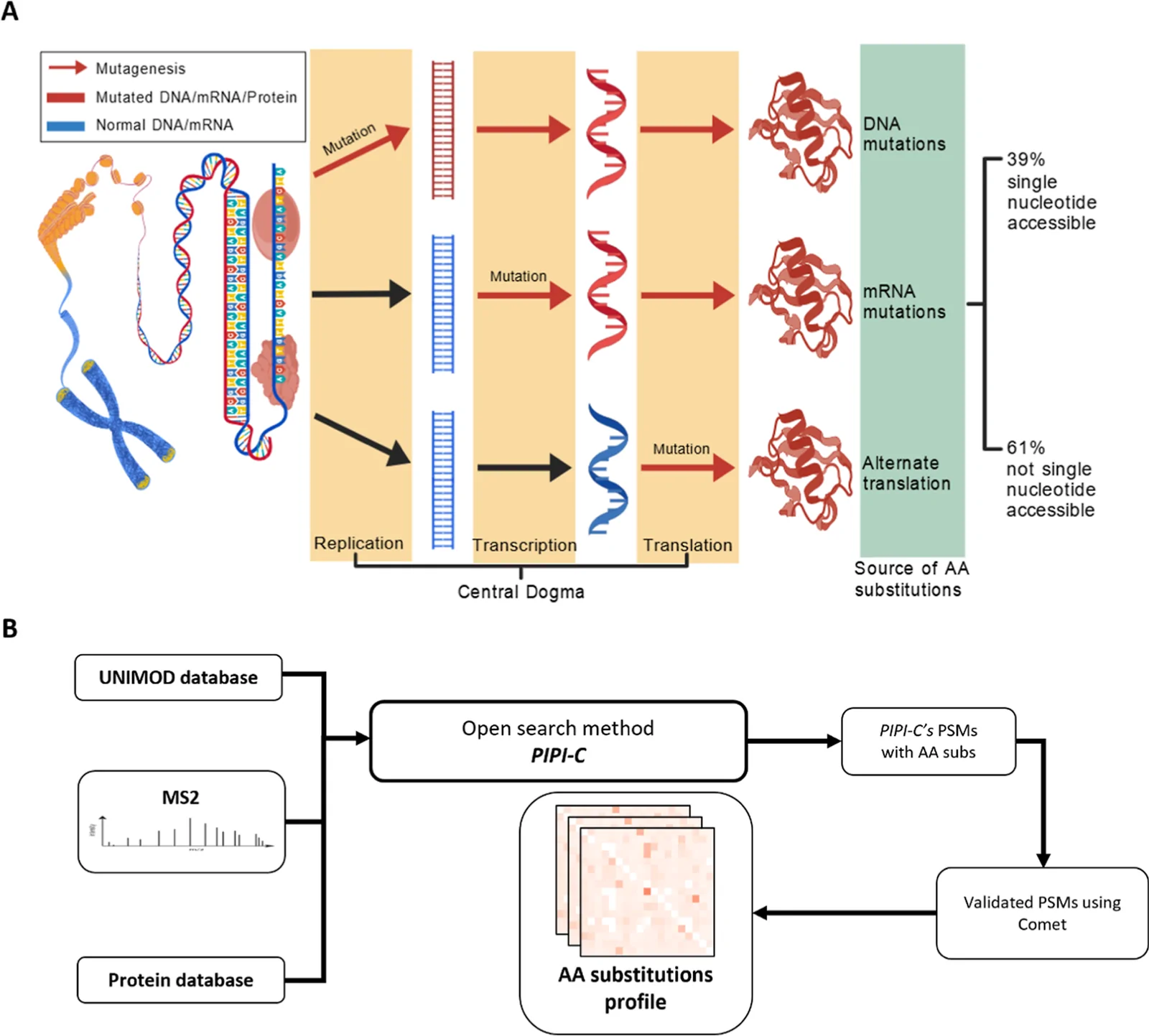
Demonstration of AA substitutomics. (A) Three sources of AA substitutions along the central dogma—DNA mutations, mRNA mutations, and alternate translation, grouped by detectability. (B) The workflow of AA substitutomics analysis.

Despite the established biological relevance of AA substitutions in cancer proteomics, comprehensive and unbiased identification of these alterations at the global proteomic scale remains a substantial technical challenge. Existing studies have been limited to targeted analyses of a narrow range of AA substitutions, leaving the complex interplay between multisite substitutions, their regulatory networks, and cancer-type specificity poorly resolved across diverse malignancies. Traditional proteomic database search strategies rely on closed-search workflows that only detect predefined post-translational modifications (PTMs) with fixed modification parameters, restricting discovery of unannotated or novel substitutions.^14,15^ While these closed-search approaches deliver high accuracy for targeted modifications, they suffer from exponential database expansion and reduced sensitivity when assessing multiple concurrent modifications, limiting their utility for global substitution profiling. This limitation has driven the development of open-search methodologies, which enable unbiased interrogation of large modification databases such as Unimod to identify uncharacterized PTMs and AA substitutions;^16^ historically, however, open-search strategies have traded search breadth for identification precision, hindering their broad clinical and research application. Recent technical advances in open-search algorithms have refined this balance, optimizing both search space coverage and identification accuracy to enable systematic, global profiling of all AA substitution types from complex proteomic data sets.^17–19^ Among these advanced tools, PIPI-C stands out for its combinatorial optimization framework for peptide identification and PTM characterization, leveraging fuzzy bidirectional matching to map short peptide sequences within protein structures and solving a mixed integer linear programming model to accurately determine peptide sequences and their corresponding PTM patterns.^17^

Here, we implement a dual-search strategy that combines the discovery capacity of open-search with the precision of closed-search, using PIPI-C as the primary open-search engine and the widely validated Comet algorithm for closed-search validation (Figure 1B). To ensure findings are robust and generalizable across diverse tumor types, we apply this optimized analytical pipeline to multiple proteomic data sets covering five distinct cancer types, minimizing tumor-specific biases in single-cohort analyses. We formally define AA substitutomics, an analytical framework for characterizing AA substitutions detected at the proteomic scale that are not captured by conventional genomic/transcriptomic variant analysis, and for dissecting the regulatory functions of key proteins carrying multiple high-significance substitutions. This AA substitutomics pipeline uncovers cancer-specific substitution signatures that correlate with distinct protein functional alterations, particularly within core oncogenic signaling pathways and protein interaction networks. In addition, this pipeline offers an approach to generating testable hypotheses about how proteomic substitutions relate to escape mechanisms in cancer.

## Materials and Methods

### Proteomic Data Sets and Data Preprocessing

In this study, proteomic data generated by the Clinical Proteomic Tumor Analysis Consortium (CPTAC) were downloaded from the Proteomic Data Commons.^20^ The analyzed data included 537 patients from five cancer cohorts: 11 TMT-11 plex experiments from 99 patients with glioblastoma multiforme (GBM, PDC000204),^21^ 20 TMT-11 plex experiments from 110 patients with head and neck squamous cell carcinoma (HNSCC, PDC000221),^22^ 25 TMT-10 plex experiments from 110 patients with lung adenocarcinoma (LUAD, PDC000153),^23^ 22 TMT-11 plex experiments from 108 patients with lung squamous cell carcinoma (LSCC, PDC000234),^24^ and 23 TMT-10 plex experiments from 110 patients with clear cell renal cell carcinoma (ccRCC, PDC000127).^25^ Drug-target-enriched data sets (ProteomeXchange PXD000788,^26^ PXD016465, PXD016505, PXD016463 ^27^) and MHC-enriched data sets (PXD003790 ^28^ and PXD000394 ^29^) were also used. All of the nonempty MS2 spectra were extracted and converted to mgf files using ProteoWizard MSConvert.^30^

This work is a computational reanalysis of previously published, publicly available, and deidentified proteomic and genomic data sets from the CPTAC and the ProteomeXchange Consortium. No new human participants or samples were recruited for this study. Ethical approval and informed consent for the original data collection were obtained by the respective consortia in their primary studies.

### Analysis of Proteomic Data

The human protein FASTA file was downloaded from UniProt^31^ (downloaded January 2024). PIPI-C was used as the open search method with the following parameters: the MS1 tolerance was 10 ppm; the MS2 tolerance was 0.01 Da; the max missed cleavage sites was 2; the enzyme was Trypsin; the peptide length was from 6 to 50; the fixed modifications were carbamidomethyl on cysteine and TMT labeling mass shift on lysine; modifications search range was ±250 Da; the variable modifications were from the Unimod,^16^ including TMT labeling mass shift on serine and n-term and all types of AA substitutions; isoleucine and leucine were treated as identical. Decoy proteins were added for false discovery rate (FDR) control with the target-decoy strategy. The remaining settings were kept as the default. Spectra were validated via Comet only when PIPI-C detected two or more replicated substitution-containing PSMs, using PIPI-C’s variable modifications and otherwise identical parameters; only PSMs identical between PIPI-C and Comet were retained.

### Details of Proteomic Quantification

In the quantification step, we only kept the PSMs that were detected with TMT labeling mass shift. The peptides with multiple PSMs were kept as quantifiable peptides. The intensities of reporter ions for each PSM were obtained from the initial MS2 spectra. Assuming unregulated peptides share comparable abundance with the common reference (CR), and thus a log TMT ratio centered at zero, we applied a two-component normalization of TMT-label intensities relative to the CR.^24^

Log ratios at the PSM level were computed as the average log values across patients within the corresponding PSM. Peptides sharing identical modification patterns were clustered into a unique PTM site pattern (UPSP). The average value of all PSM-level TMT ratios in each UPSP was used as the UPSP ratio. Each cancer cohort was composed of a set of distinct experiments. Therefore, the distinct average log ratios for each UPSP in each experiment were calculated. We only selected those UPSPs that appeared in over 3/5 of the experiments for further analysis and quantification. Differential abundance was assessed with a moderated *t*-test (limma with empirical-Bayes variance shrinkage) and BH–FDR correction. The multiplexed TMT design was accounted for at the quantification stage: for each UPSP, a within-plex tumor-to-normal ratio was computed relative to the common reference channel of the same plex, and the per-UPSP value was taken as the mean of these within-plex contrasts across experiments. As a robustness check for the multiplexed design, each UPSP was additionally refitted with a linear mixed-effects model in which log2 abundance was modeled as a function of the tumor-versus-normal condition (fixed effect), with random intercepts for TMT plex and patient; the resulting effect sizes were highly concordant with the limma estimates, confirming that the findings are robust to the modeling choice (full comparison in Supplementary Data S11).

### GO and KEGG Enrichment Analysis

Proteins that had been quantified (in quantification results, *q*-value < 0.05 and fold change > 3 or fold change < 1/3) and UPSPs with AA substitution modifications were subjected to GO enrichment and KEGG enrichment analyses, where the background was the set of proteins identified in the corresponding cohort (5838–7202 proteins per cohort), rather than the whole human proteome. Two-tailed Fisher’s exact test and BH-FDR correction were used to measure the statistical significance of the enrichment analyses. The visualizations in this study only included the enrichment terms with q-values less than 0.01 in GO and KEGG analysis. The script for GO enrichment analysis was developed using GOATOOLS,^32^ while the KEGG pathway enrichment analysis was conducted using GSEApy.^33^

### Terminology and Construction of Protein–Protein Interaction Network

We incorporated PPI data sourced from the STRING database,^34^ which aggregates interaction information from various sources such as text mining, experiments, databases, coexpression, neighborhood, gene fusion, and co-occurrence. The PPI network was visualized using Cytoscape (version 3.10.2).^35^ In this network representation, each pair of interacting proteins and the link between them were respectively denoted as nodes and edges.

### Protein Structure Modeling, Comparing, and Measuring

The protein structures were aligned by the Matchmaker function of ChimeraX (version 1.4).^36^ The structures were visualized with ChimeraX. The structure of the Filamin-A (FLNA) Ig-like domains 3–5 was obtained by X-ray diffraction from *Escherichia coli* expression (PDB: 4M9P). The X-ray diffraction structure of vimentin (fragment 144–251) was from humans (PDB: 3UF1). The structure of human kidney fructose-bisphosphate aldolase B (ALDOB) was obtained by cryo-electron microscopy (PDB: 8D44). AlphaFold3 built the predicted structures with cumulative AA substitutions on the original sequences. When there was more than one possible AA substitution identified on the same site, we preferred the AA substitution with the higher regulation fold change. The predicted structure with the best pLDDT score was used for structure comparison and similarity measurement. The model incorporates all substitutions detected in this protein simultaneously and is shown only to illustrate potential cumulative structural effects; it does not represent a predominant proteoform, as individual substitutions occur at low stoichiometry across a heterogeneous population of protein molecules.

### Quantification of Binding Affinity between the Drug-Target Pairs

Binding affinities were predicted using CB-Dock2.^37^ The uploaded structures included both the wild-type drug targets from the PDB database and the AlphaFold3 predicted structures with AA substitutions. All proteins were uploaded in PDB format. The ligand used in CB-Dock2 was obtained from the DrugBank database,^38^ also in PDB format. To compare changes in binding affinity, we selected the results that shared the same docking size and selected the results with the lowest Vina score. All other settings were maintained at their default values in CB-Dock2.

### MHC Binding Affinity Prediction

The predictions for MHC binding affinity and the count of MHC binders were generated using NetMHCpan 4.2.^39^ Both the wild-type sequences and the sequences modified by AA substitutions were submitted in fasta format to the web-based interface of NetMHCpan. The representative from the human leukocyte antigen (HLA) supertype was selected for the predictions. All other settings were maintained at their default values, with the binder threshold set at 2%, indicating that peptides predicted to fall within the top 2% of binding affinities are classified as predicted binders. The binders with the top 0.5% of binding affinities were regarded as strong binders.

To quantify the impact of AA substitutions in MHC proteins, we gathered the presented peptide sequences from the PDB database for HLA-A, HLA-B, and HLA-C, respectively. We then entered the wild-type protein sequences along with the AA substitution-modified sequences as custom MHC protein sequences in NetMHCpan 4.2 to predict and compare their binding affinities.

### Site- and Peptidoform-Level Validation

Substitution evidence was aggregated from peptide-spectrum matches to peptidoforms and to substitution events. A substitution site was defined as a residue position (protein and absolute residue position) at which one or more substitutions are detected, and a substitution event (or distinct substitution) as a specific substituted AA at that position; absolute positions were obtained by aligning each peptide’s bare sequence to its UniProt protein sequence (isoleucine and leucine treated as equivalent) and adding the within-peptide offset of the substituted residue, which was determined by matching the monoisotopic mass difference of the reported substitution (±0.02 Da). PSMs selected for quantification were required to recur across replicates, as described above. Residue-level accuracy was evaluated against the ground truth of the simulated data sets of Supplementary Note 3, which record the true substituted positions. A spectrum was scored correct at the peptidoform level only if the reported bare peptide sequence matched the true sequence exactly, and at the residue level only if every true substitution on that spectrum was localized to the exact position; all four engines were scored identically at 1% FDR. Cross-study reproducibility of substitution events was assessed between the two lung-cancer cohorts, which derive from independent patient series and independent TMT batches. Isobaric ambiguity was assessed by comparing each substitution’s monoisotopic mass difference with the modification patterns of Supplementary Table S4, requiring both mass equality within 0.01 Da and Unimod annotation of the modification on the residue.

### Genomic and RNA-Editing Cross-Validation

Matched somatic mutation calls for the same patients were obtained through the CPTAC Python package.^40^ For each identified substitution, all codons of the reference and observed AA were enumerated, and the substitution was recorded as single-nucleotide accessible if any pair differed at exactly one position; the identity of that base change (e.g., A > G, C > T) was retained to assess consistency with A-to-I and C-to-U RNA editing. This cross-reference used matched somatic mutation calls only; single-nucleotide-accessible substitutions may stem from undetected low-frequency variants, and germline coding variants were not evaluated separately.

## Results

### AA Substitution Landscape Across Five Cancer Cohorts

Building on the pipeline (Figure 1), we first benchmarked PIPI-C,^17^ whose open-search engine is described in detail in Supplementary Note 1, against three state-of-the-art open-search engines (Open-pFind,^18^ MODplus,^19^ and MSFragger,^41^) on simulated and synthetic-peptide data sets, assessing false-positive control (Supplementary Note 2), detection sensitivity and residue-level localization accuracy against ground truth (Supplementary Note 3), and multimodification capacity (Supplementary Note 4). PIPI-C showed a low false-identification rate (Figures S2, S3). For single substitutions it was not the most accurate engine, but the engines diverged sharply when substitutions co-occurred: scored against the true positions, PIPI-C localized 84.2%, 68.0%, and 51.1% of substitution-bearing spectra for one, two, and three substitutions per peptide, whereas the best competitor fell to 48.8% at two and 23.1% at three (Supplementary Note 3, Supplementary Data S9). Therefore, PIPI-C is exploited as the most suitable engine that retains both detection and residue-level assignment when multiple substitutions co-occur within one peptide, which is the technical prerequisite for unbiased AA substitutomics. It also distinguishes biologically enriched substitutions from nonspecific background (Supplementary Note 5).

In this study, we profiled global AA substitutions across five CPTAC cancer cohorts^42^ (537 patients; per-cohort counts and accessions in Methods). These five cancer types were selected for comparable cohort sizes (99–110 patients), uniform isobaric TMT-10/11 acquisition, and anatomical diversity. The overall proteomic data analysis workflow is outlined in Figure 1B. AA substitutions identified by PIPI-C were subsequently validated with the Comet closed-search algorithm, and only cross-validated results were retained for downstream profiling and analysis. Both PIPI-C and Comet were run with a PSM-level FDR cutoff of 1% to ensure result quality. We also identified potential identification ambiguities stemming from identical mass shifts between specific AA substitutions and other PTMs (Supplementary Table S4), and all such ambiguous substitutions were excluded from subsequent analyses to eliminate false positive calls. We confirmed that no reported substitution is isobaric with a modification annotated on its own residue. Substitution evidence was further aggregated from PSMs to peptidoforms and to substitution events: every reported substitution is supported by 7 to 16 PSMs across 7 to 16 independent TMT experiments, and 922 substitution events are reproduced identically (same protein, residue, and substituted AA) between the two independent lung-cancer cohorts (Figure S7, Supplementary Data S10). Detailed search results of the AA substitutomics pipeline are presented in Figure S8.

Normalized ratios of individual AA substitution types across the five cancer cohorts are visualized as heatmaps in Figure 2A, revealing several conserved pan-cancer substitution patterns. Notably, among all glutamic acid-derived substitutions (E > X), the E > M substitution was the most abundant, accounting for 23–28% of all E-related substitutions across cohorts. Similarly, among proline-derived substitutions (P > X), the P > V-substitution was the most prevalent, representing 25–39% of total P-related substitutions (Supplementary Data S1). Both PIPI-C identification results and Comet-validated results are available on Zenodo^43^ under record number 13383150.

**Figure 2:**
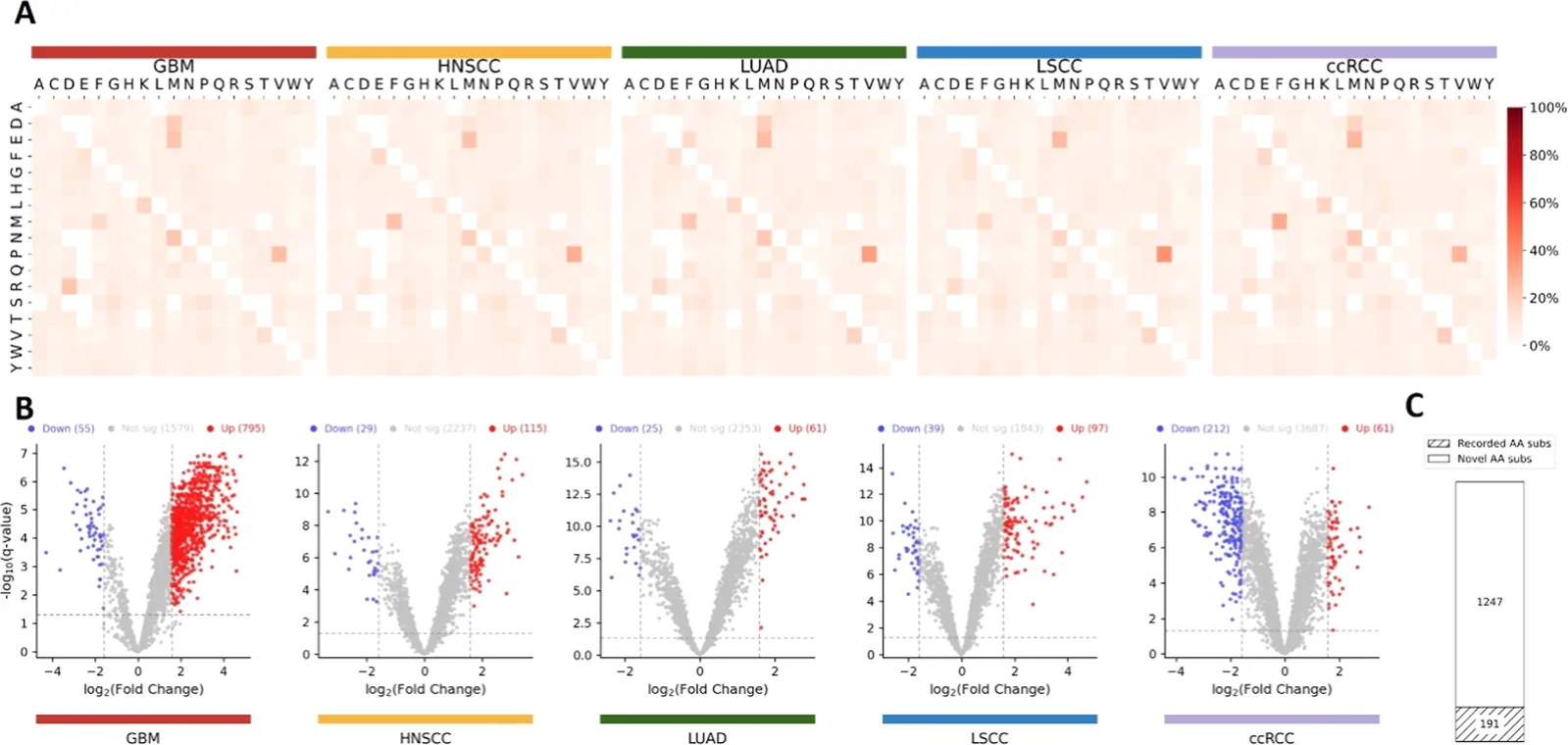
AA substitution landscape across five cancer cohorts. (A) Heatmaps show the normalized ratios of all types of AA substitutions in validated PSMs among five cancer cohorts. For each cohort, the ratio of a given substitution type is normalized within its source residue—the number of substitutions of that type divided by the total number of substitutions originating from the same initial AA (each row sums to 1). The *y*-axis AAs are the initial AAs, and the *x*-axis AAs are the substituted AAs; the shade of red represents this within-source normalized ratio. Cohort colors are used throughout: GBM, dark red; HNSCC, yellow; LUAD, green; LSCC, blue; ccRCC, purple (Supplementary Data S1). For the search results of the AA substitutomics pipeline, please refer to Figure S8. (B) Volcano plots of quantification results based on the validated results. Downregulated UPSPs are denoted by blue. Upregulated UPSPs are denoted by red. Not significantly regulated UPSPs are denoted by gray (Supplementary Data S2). (C) Bar plot of the proportion of identified AA substitutions recorded in the protein variant database (Supplementary Data S3). Clean: substitutions not recorded in the variant database; slashed: recorded substitutions.

We next performed quantitative profiling to assess differential regulation of AA-substitution-containing UPSPs between tumor and matched normal tissue samples (Figure 2B, Supplementary Data S2). Regulation profiles of UPSPs carrying all modification types are shown in Figure S9. Quantitative analysis revealed that the majority of AA substitution-modified UPSPs were not significantly differentially regulated between tumor and normal samples. Among significantly regulated UPSPs, only 13% (191 out of 1438) of the identified AA substitutions were annotated in the UniProt protein variant database,^31^ while the remaining 1247 AA substitutions were not recorded (Figure 2C, Supplementary Data S3). In addition, cross-referencing all 13,399 identified substitutions against the matched CPTAC whole-exome data of the same patients yielded no matches to patient-specific somatic mutations: 61% require two or more base changes and cannot be produced by any single-nucleotide variant, making them identifiable only by direct proteome-scale profiling, whereas the remaining 39% are single-nucleotide-accessible, for which an undetected low-frequency DNA/RNA variant cannot be excluded (Figure S17, Supplementary Data S13).

Notably, AA substitutions were unevenly distributed across the proteome in all cancer cohorts. Hemoglobin subunit beta (HBB) was identified as one of the proteins with the highest number of AA substitutions, with a total of 104 distinct substitutions detected across the five cohorts, despite its short AA length of only 147 residues (Figure S10); consistent with pan-cancer patterns, the E > M substitution remained the most abundant substitution in HBB. Additional proteins harboring multiple significantly regulated AA substitutions are listed in Figure S11. Differential regulation patterns varied markedly across cancer types: in four cohorts (GBM, HNSCC, LUAD, and LSCC), the number of upregulated UPSPs exceeded that of downregulated UPSPs, with GBM exhibiting the highest number of upregulated UPSPs (795). In stark contrast, ccRCC displayed an inverse regulatory pattern, with significantly more downregulated UPSPs (212) than upregulated UPSPs (61). These distinct cohort-specific quantitative profiles highlight pronounced cancer type-specific AA substitution regulation, which we further interrogated in subsequent focused analyses of significantly regulated UPSPs.

### Multiple Significantly Regulated AA Substitutions in Cancer-Associated Proteins

Our AA substitutomics pipeline identified eight candidate cancer-associated proteins harboring multiple significantly dysregulated AA substitutions. They provide a high-priority candidate pool for future functional and translational study of protein-level variations not captured by genomic profiling in cancer. These findings reflect only statistical associations and require dedicated clinical validation. We performed GO and KEGG pathway enrichment analyses^44,45^ and describe below the substitutions in proteins related to extracellular exosomes, actin filament binding, and glycolysis/gluconeogenesis; detailed per-protein descriptions for all three themes are provided in Supplementary Note 7.

### AA Substitutomes in Extracellular Exosome Proteins Across Cancer Cohorts

Extracellular exosomes are small (<100 nm) cell-secreted vesicles that mediate intercellular communication and have been repeatedly implicated in cancer progression and the tumor microenvironment.^46–48^ In this study, GO enrichment analysis revealed that the term extracellular exosome was significantly enriched across all five cohorts (Figure S12). Among cellular component terms, three of the top eight were associated with the extracellular compartment (Figure 3A), suggesting a potential association between AA substitutions and the tumor extracellular microenvironment. The counts of exosome-related genes per cancer type are displayed in Figure 3B. GBM had the highest number of exclusively upregulated exosome-related proteins, exceeding the other four cancer types; these counts were 86, 32, 36, 43, and 11 in GBM, HNSCC, LUAD, LSCC, and ccRCC, respectively.

**Figure 3:**
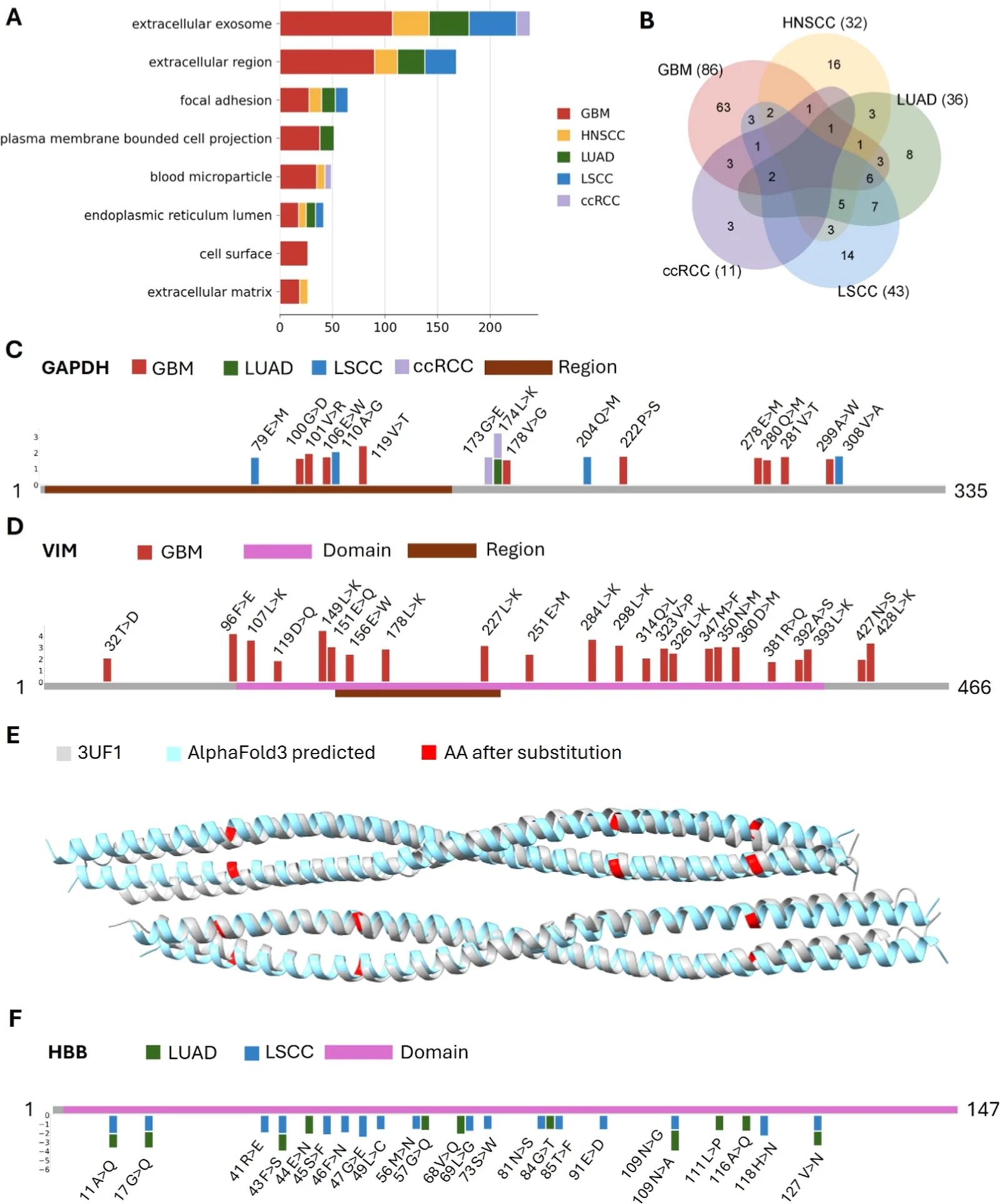
AA substitutomes in extracellular exosome proteins across cancer cohorts. (A) Bar plot showing the cellular component terms of GO enrichment. (B) Protein overlap of only upregulated genes. The number of only upregulated genes in each cohort is noted. (C) Cumulative AA substitution map of GAPDH across different cancer cohorts. The AA substitutions above the protein sequence are upregulated. Barplot showing the base 2 logarithm ratio of cancer regulation (Supplementary Data S4). Brown segment: binding and interaction region. (D) Cumulative AA substitution map of VIM in GBM. The AA substitutions above the protein sequence are upregulated. Barplot shows the base 2 logarithm ratio of cancer regulation (Supplementary Data S4). Brown segment: binding and interaction region. Pink segment: structural domain. (E) Structure comparison between the X-ray diffraction structure of human vimentin (fragment 144–251) (PDB: 3UF1) and structure prediction with cumulative AA substitutions. Gray: the structure of 3UF1. Light blue: AlphaFold3-predicted structure with cumulative AA substitutions in fragments 144–251, which does not correspond to a predominant proteoform. Red: The AAs after substitution. (F) Cumulative AA substitution map of HBB in two types of lung cancers. The AA substitutions below the protein sequence are downregulated. Barplot showing the base 2 logarithm ratio of cancer regulation (Supplementary Data S4). Pink segment: structural domain.

We characterized three key exosome-associated proteins—glyceraldehyde 3-phosphate dehydrogenase (GAPDH), vimentin (VIM), and HBB (Figure 3C,D,F); detailed residue-level descriptions are in Supplementary Note 7. GAPDH carried ten substitutions in GBM (and one in LUAD, four in LSCC, two in ccRCC), several within its WARS1-binding region (residues 2–148).^49^ VIM had 23 upregulated sites—including two events at site 251 (E251M, 16-fold; E251F, 5.54-fold)—with 19 sites in the intermediate-filament rod domain (residues 103–411); an AlphaFold3 model of the coiled-coil region (residues 154–245; modeled over fragment 144–251, carrying E251M) illustrates potential cumulative structural effects rather than a predominant proteoform (Figure 3E).^50,51^ As the representative example, HBB carries one of the highest substitution densities of any protein: its substitutions are downregulated exclusively in the two lung-cancer cohorts—17 downregulated substitutions across 15 sites in LSCC and 11 in LUAD, all within the globin domain (residues 3–147; Figure 3F)—including the shared A11Q, G17Q, and F43S and the known DNA-derived E44K and M56I (sites 44 and 56).^52^ Together with seven downregulated hemoglobin subunit alpha 1 (HBA1) UPSPs in LSCC, this marks a lung-cancer-specific signature. Two HBB sites are literature-validated—F43S, which alters the heme-pocket configuration and destabilizes hemoglobin,^53–55^ and E91D, a documented high-oxygen-affinity variant whose downregulation is consistent with reduced HBB oxygen affinity in the hypoxic lung-tumor microenvironment.^56–59^ The consistent downregulation of these two functionally opposite HBB substitutions specifically in lung cancers points to a coordinated, disease-specific remodeling of hemoglobin in the hypoxic tumor microenvironment. The annotated MS/MS spectra for these and six other substitutions are provided in Figure S13.

### AA Substitutomes in Actin Filament Binding Proteins Across Cancer Cohorts

Actin filaments are central cytoskeletal structures whose dynamics are governed by actin-binding proteins, which have been widely linked to cancer progression and therapeutic opportunities.^60–63^ Across the five cohorts, GO molecular-function enrichment against the detected proteome yielded significant terms including actin filament binding, one of the most specific cytoskeletal terms; GBM contributed the most enriched molecular-function terms overall (12), followed by ccRCC (5); the actin-filament-binding term itself is significant in GBM, HNSCC, LUAD and LSCC but not ccRCC (Figure 4A). Because AA substitutions can alter a protein’s primary structure, and hence its fold and intramolecular bonding, they may in turn affect binding sites and function.^64^ The GO molecular function enrichment results based on the downregulated UPSPs are shown in Figure S14.

**Figure 4:**
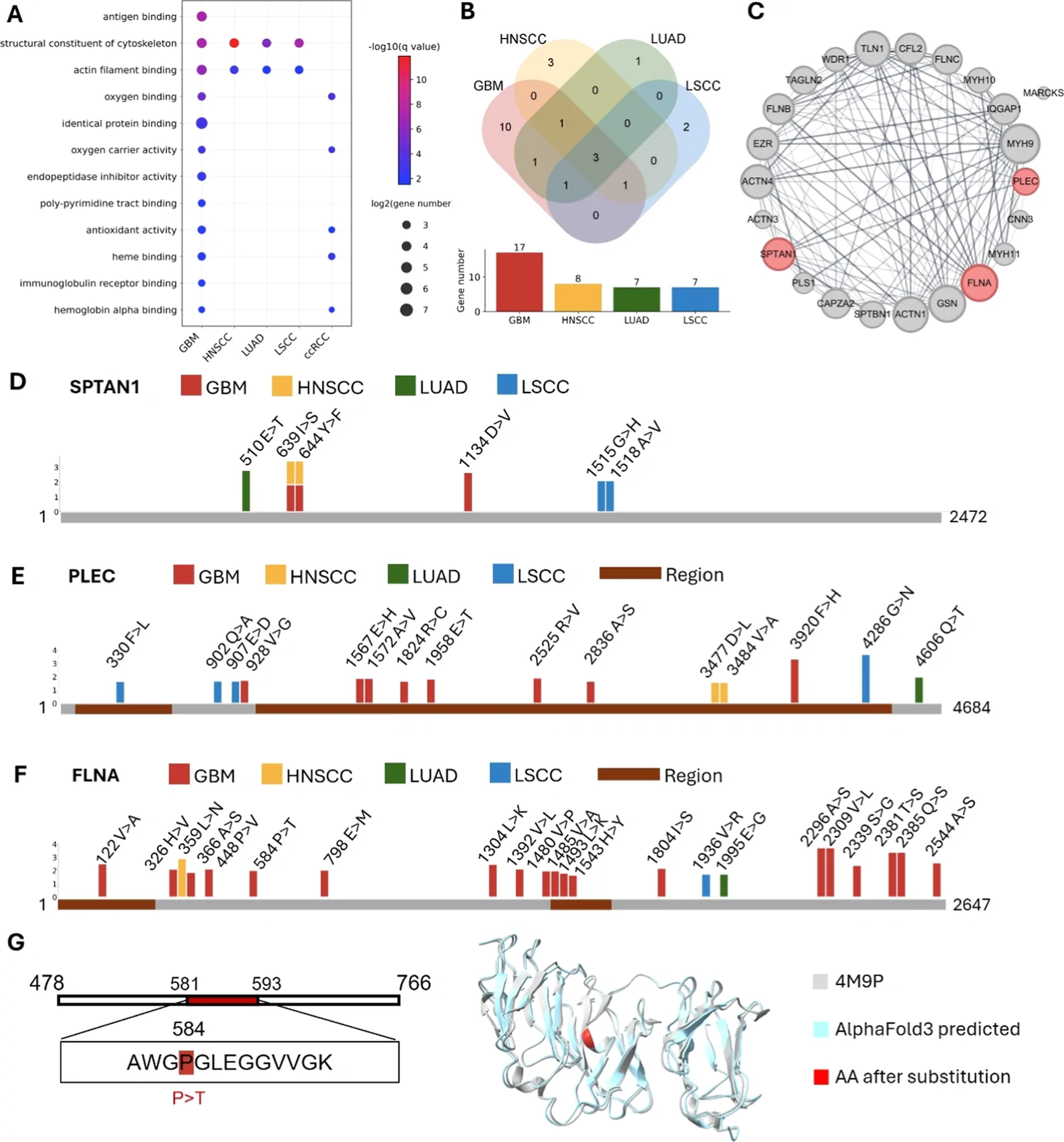
AA substitutomes in actin filament binding proteins across cancer cohorts. (A) Bubble plot showing the molecular function terms of GO enrichment based on the upregulations in the five cancer cohorts. The size of the bubble represents the base 2 logarithm of the enriched gene number, and the color of the bubble shows the minus base 10 logarithm of the *q*-value. (B) Venn plot (top) and bar plot (bottom) demonstrating the number of genes related to actin filament binding in four cohorts of cancers. (C) Diagram representing a network of 23 proteins corresponding to actin filament binding that are significantly upregulated. The size of the node represents the degree of the protein. (D) Cumulative AA substitution map of SPTAN1 across different cancer cohorts. The AA substitutions above the protein sequence are upregulated (Supplementary Data S4). Barplot showing the base 2 logarithm ratio of cancer regulation. (E) Cumulative AA substitution map of PLEC across different cancer cohorts. The AA substitutions above the protein sequence are upregulated (Supplementary Data S4). Barplot showing the base 2 logarithm ratio of cancer regulation. Brown segment: binding and interaction region. (F) Cumulative AA substitution map of FLNA across different cancer cohorts. The AA substitutions above the protein sequence are upregulated (Supplementary Data S4). Barplot showing the base 2 logarithm ratio of cancer regulation. Brown segment: binding and interaction region. (G) Structure comparison between the X-ray diffraction structure of FLNA Ig-like domains 3–5 (PDB: 4M9P) and structure prediction with AA substitution of P584T from the GBM cohort. Left: the site of proline-to-threonine (P584T) substitution in the protein sequence. Right: the structure of 4M9P (gray) and the AlphaFold3-predicted structure after P584T substitution (light blue). The AAs after substitution (red).

The number of actin filament binding-related genes per cohort and their overlap are shown in Figure 4B (colors denote four cancer types, ccRCC excluded). Among the four cancers, 23 proteins are actin filament binding-related, with GBM contributing 17; the protein–protein interactions among these 23 proteins are displayed in Figure 4C, where node size reflects each protein’s degree.

We detected substitutions in SPTAN1, plectin (PLEC), and filamin-A (FLNA) (Figure 4D–F); detailed residue-level descriptions are in Supplementary Note 7. SPTAN1 (spectrin α-chain) carried the common I639S and Y644F substitutions in GBM and HNSCC (3.56- and 3.07-fold).^65,66^ SPTAN1 has context-dependent roles in tumor progression, so these shared scaffold substitutions may influence cytoskeletal organization. PLEC carried a relatively large number of substitutions (eight upregulated in GBM), spanning an actin-binding region (residues 175–400) and an IF-interaction region (residues 964–4574).^67–69^ Because nearly all covered PLEC substitutions are upregulated and cluster in the intermediate-filament–interaction region, the plectin–IF linkage may be selectively perturbed in GBM. As a representative example, FLNA links actin filaments to membrane integrins and drives GBM proliferation and invasion,^70–73^ carrying upregulated substitutions at 19 sites in GBM (Figure 4F). Among these, we highlight P584T in Ig-like domain 4—a hydrophobic-to-hydrophilic change with 4-fold upregulation—whose side chain orients toward the β-sheet hydrophobic core and is predicted to perturb core packing and the FLNA–integrin interface.^74–76^ We modeled the P584T variant with AlphaFold3 ^77^ against the FLNA Ig-like domains 3–5 X-ray structure (PDB: 4M9P; Figure 4G).

### AA Substitutomes in Glycolysis and Gluconeogenesis Proteins Across Cancer Cohorts

Glycolysis and gluconeogenesis are reciprocally regulated pathways that are central to the rapid energy demands of proliferating cancer cells, and their dysregulation has been linked to therapeutic vulnerabilities.^78–80^ KEGG pathway enrichment^45^ among the five cancer cohorts shows that the AA substitution-containing proteins are enriched in the pathways of glycolysis and gluconeogenesis. Among upregulated substitutions, four cohorts (all except HNSCC) were enriched in these pathways (Figure 5A, arrow), whereas among downregulated substitutions, only ccRCC was enriched (Figure 5B, arrow).

**Figure 5:**
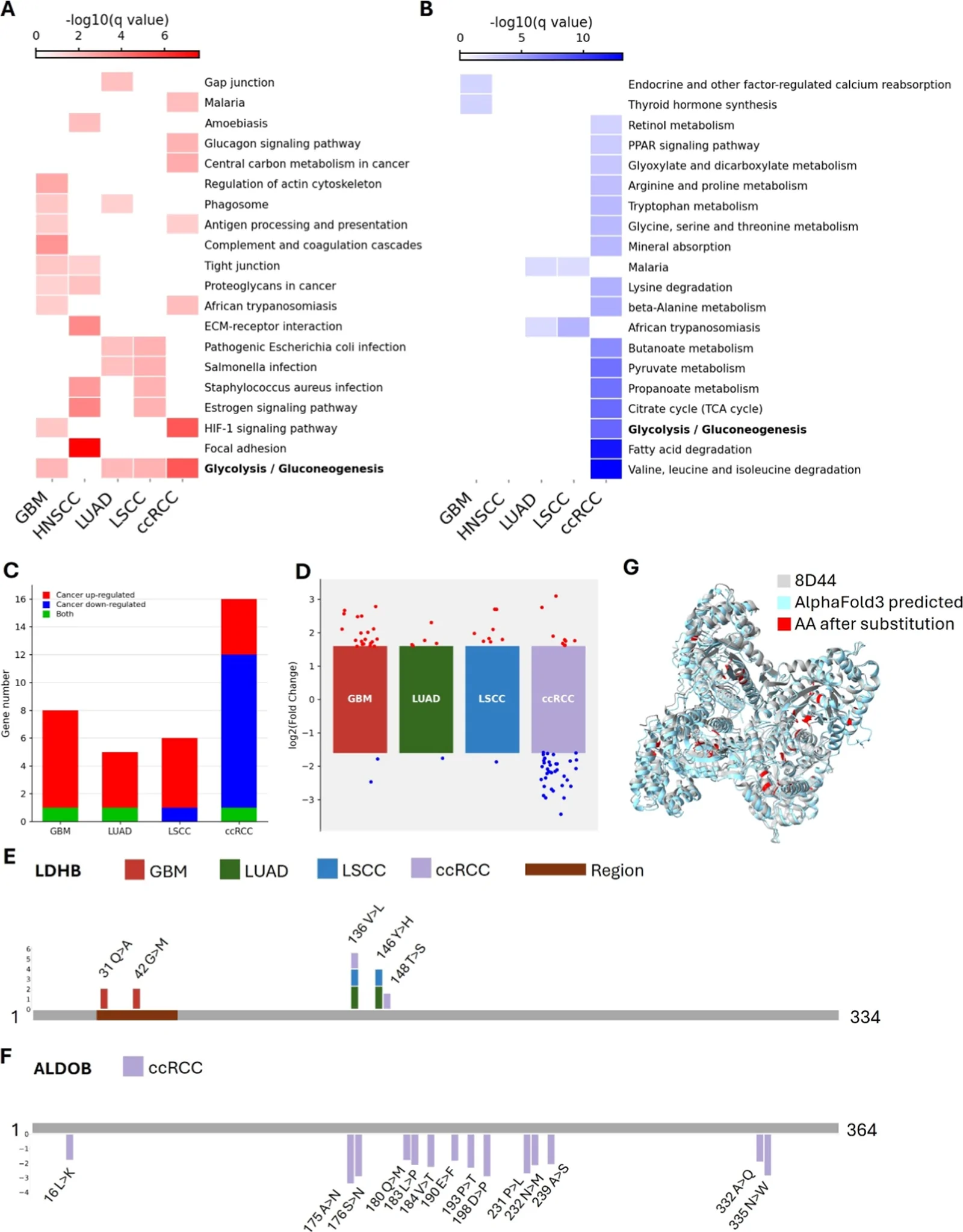
AA substitutomes in glycolysis and gluconeogenesis proteins across cancer cohorts. (A) Heatmaps showing KEGG enrichment results of upregulations among five cancer cohorts. The color represents the minus base 10 logarithm of the *q*-value; the darker the red, the smaller the *q*-value. (B) Heatmaps showing KEGG enrichment results of downregulations among five cancer cohorts. The color represents the minus base 10 logarithm of the *q*-value; the darker the blue, the smaller the *q*-value. (C) Bar plot showing the number of genes related to glycolysis and gluconeogenesis among four types of cancers. The upregulated-only genes are denoted by the red segment, the downregulated-only genes are denoted by the blue segment, and the both up- and downregulated genes are denoted by the green segment. (D) Volcano plot presenting the gene expression of glycolysis and gluconeogenesis corresponding to UPSPs. (E) Cumulative AA substitution map of LDHB in four types of cancers. The AA substitutions above the protein sequence are upregulated (Supplementary Data S4). Barplot showing the base 2 logarithm ratio of cancer regulation. Brown segment: binding and interaction region. (F) Cumulative AA substitution map of ALDOB in ccRCC. The AA substitutions below the protein sequence are downregulated (Supplementary Data S4). Barplot showing the base 2 logarithm ratio of cancer regulation. (G) Structure comparison between the cryo-electron microscopy structure of human renal ALDOB (PDB: 8D44) and structure prediction with cumulative AA substitutions from ccRCC. Gray: the structure of 8D44. Light blue: AlphaFold3-predicted structure with cumulative AA substitutions, which does not correspond to a predominant proteoform. Red: The AAs after substitution.

The numbers of glycolysis and gluconeogenesis pathway-related genes are displayed in Figure 5C. Among the four cohorts, GBM has seven upregulated genes, which is more than the other cancer types, while ccRCC has 11 downregulated genes and one bidirectionally regulated gene, and is the only cancer in which downregulated AA substitutions predominate. The regulatory situation of the significantly regulated UPSPs of the four cohorts is demonstrated in Figure 5D. In our analysis, the l-lactate dehydrogenase B chain (LDHB) was identified in common across all four cancers (GBM, LUAD, LSCC, and ccRCC). LDHB, a key glycolytic enzyme interconverting pyruvate and lactate, is actively regulated in cancer cells.^81,82^ While the critical involvement of LDHB in the advancement of different cancers has been increasingly documented,^83,84^ the AA substitutomics analysis of LDHB across different cancers remains inadequate. ALDOB, a glycolytic aldolase,^85^ carries the largest number of AA substitutions among the downregulated proteins. ALDOB downregulation is a candidate prognostic biomarker in ccRCC and has been linked to its clinicopathological characteristics.^86^ The literature validates the findings in this study of downregulated AA substitutions in ALDOB from the ccRCC cohort.

Detailed residue-level descriptions of LDHB and ALDOB are provided in Supplementary Note 7 (Figure 5E–G). Some substitution sites correspond to documented DNA-mutation-derived substitutions—V136M in LDHB, and P231R and A239S in ALDOB.^52^ LDHB carried two upregulated substitutions per cohort, including two in the NAD^+^ binding region (residues 31–53) in GBM that could interfere with pyruvate–lactate interconversion.^87^ LDHB is increasingly implicated in aggressive cancer phenotypes and lysosomal/autophagic activity, making these NAD^+^-region substitutions of potential functional interest. As a representative example, ALDOB carries an A175N-substitution at a well-documented substitutional hotspot^88–90^ within an α-helical region proximal to substrate-binding residues, where the A > N change may perturb local packing and an intrachain hydrogen bond (residues 175–179)^91,92^ (Figure 5F); all identified ALDOB substitutions occur exclusively in the ccRCC cohort with consistent downregulation, aligning with the reported link between ALDOB downregulation and ccRCC pathology.^86^ To visualize the structural impact, we compared the cryo-EM structure of human kidney ALDOB (PDB: 8D44) with an AlphaFold3 model carrying the cumulative substitutions (Figure 5G), which does not correspond to a predominant proteoform. Functional characterization of these substitutions remains to be performed.

### AA Substitutomics Identifies Candidate Substitutions in Drug-Escape–Associated Proteins

AA substitutions in metabolic enzymes and transporters can contribute to cancer drug escape by perturbing drug binding while preserving activity.^93–97^ Our pipeline identifies four AA substitutions in low-density lipoprotein receptor-related protein 2 (LRP2, also known as megalin) in the ccRCC cohort (Supplementary Data S2 and Figures 6A, S15). Because the current structural model (PDB: 9CWM) is discontinuous, we compared the experimental and AlphaFold3-predicted structures over segments 3501–3700 and 4201–4300 spanning the substitutions (Figure 6B); the substitutions appear to have a modest structural effect. As LRP2 is under investigation as a therapeutic-antibody target,^98,99^ substitutomics profiling of LRP2 may inform strategies that circumvent associated drug-escape mechanisms. We also identify LDHB as potentially involved in drug escape.^100^ We identified five AA substitutions across four cohorts (GBM, LUAD, LSCC, ccRCC) that may affect LDHB structure. In the crystal structure of the LDHB-NADH-oxamic acid complex,^101,102^ asparagine 138 is a key binding residue; the pipeline reveals a nearby V136L substitution predicted to affect LDHB binding affinity (Figure 6C). Although NADH and oxamic acid are not cancer drugs, NADH metabolism and oxamic acid have recognized roles in cancer-cell treatment and as anticancer leads.^103,104^

**Figure 6:**
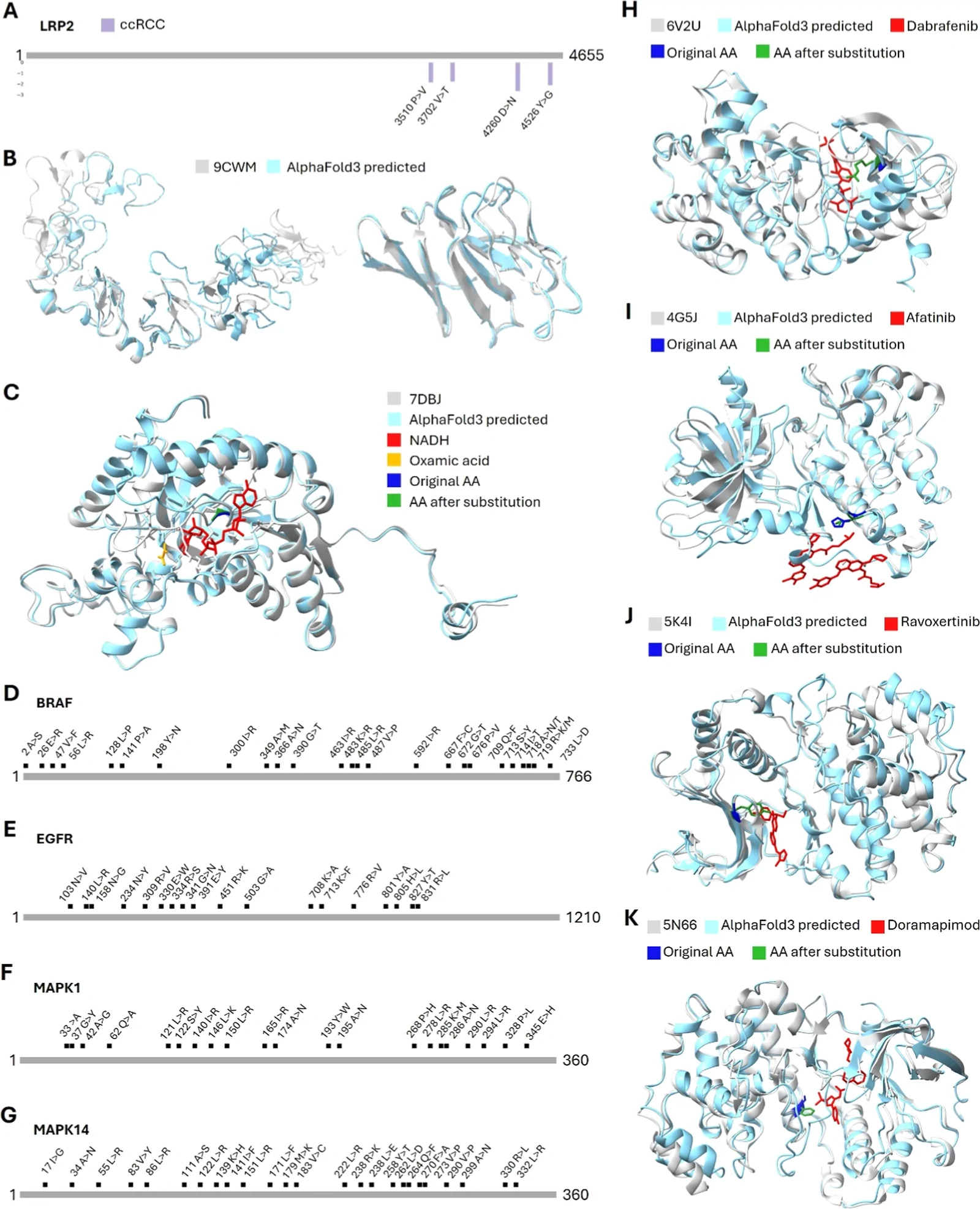
AA substitutomics highlights candidate drug-escape–associated substitutions. (A) Cumulative AA substitution map of LRP2 in the ccRCC cohort. The AA substitutions below the protein sequence are downregulated. Barplot showing the base 2 logarithm ratio of cancer regulation (Supplementary Data S2). (B) The structure comparison between the cryo-electron microscopy structure of human LRP2 (PDB: 9CWM) and the structure prediction of LRP2 in the segment of 3501–3700 (left) and 4201–4300 (right). Gray: the structure of 9CWM. Light blue: AlphaFold3-predicted structure with cumulative substitutions, which does not correspond to a predominant proteoform. (C) The structure comparison between the X-ray diffraction structure of human LDHB (PDB: 7DBJ) and the structure prediction of LDHB. Gray: the structure of 7DBJ. Light blue: AlphaFold3-predicted structure with cumulative substitutions, which does not correspond to a predominant proteoform. Red: NADH. Orange: oxamic acid. Deep blue: the original AA. Green: the AA after substitution. (D) Cumulative AA substitution map of BRAF (Supplementary Data S5). (E) Cumulative AA substitution map of EGFR (Supplementary Data S5). (F) Cumulative AA substitution map of MAPK1 (Supplementary Data S5). (G) Cumulative AA substitution map of MAPK14 (Supplementary Data S5). (H) Structure comparison between the X-ray diffraction structure of human BRAF (PDB: 6V2U) and the structure prediction of BRAF. Gray: the structure of 6V2U. Light blue: AlphaFold3-predicted structure with cumulative substitutions, which does not correspond to a predominant proteoform. Red: Dabrafenib. Deep blue: the original AA. Green: the AA after substitution. (I) Structure comparison between the X-ray diffraction structure of human EGFR (PDB: 4G5J) and the structure prediction of EGFR. Gray: the structure of 4G5J. Light blue: AlphaFold3-predicted structure with cumulative substitutions, which does not correspond to a predominant proteoform. Red: Afatinib. Deep blue: the original AA. Green: the AA after substitution. (J) Structure comparison between the X-ray diffraction structure of human MAPK1 (PDB: 5K4I) and the structure prediction of MAPK1. Gray: the structure of 5K4I. Light blue: AlphaFold3-predicted structure with cumulative substitutions, which does not correspond to a predominant proteoform. Red: Ravoxertinib. Deep blue: the original AA. Green: the AA after substitution. (K) Structure comparison between the X-ray diffraction structure of human MAPK14 (PDB: 5N66) and the structure prediction of MAPK14. Gray: the structure of 5N66. Light blue: AlphaFold3-predicted structure with cumulative substitutions, which does not correspond to a predominant proteoform. Red: Doramapimod. Deep blue: the original AA. Green: the AA after substitution.

Many proteins have been identified as drug targets in cancer therapeutics, including serine/threonine-protein kinase B-raf (BRAF), epidermal growth factor receptor (EGFR), and mitogen-activated protein kinase (MAPK) proteins.^105^ Because such targets were not abundant across the five cohorts relative to the eight proteins discussed earlier (Figure S15), we additionally analyzed enriched drug-target data sets from ProteomeXchange (PXD000788,^26^ PXD016465, PXD016505, and PXD016463;^27^ PIPI-C results on Zenodo, record 17669590). These data sets were generated to study interactomes and signaling networks rather than drug-resistant tumors, so the identified substitutions represent hypothetical candidates for potential resistance rather than observations in confirmed drug-resistant states. Figure 6D–G maps the cumulative substitutions for BRAF, EGFR, MAPK1, and MAPK14, and Figure 6H–K compares wild-type and AlphaFold3-predicted substituted structures. Docking of each drug–target pair before and after substitution was compared with CB-Dock2 (Vina score; lower score means stronger binding) for qualitative, hypothesis-generating purposes only, as detailed in the Materials and Methods.

These four kinases are frontline oncology targets whose inhibitors are in clinical use, so substitutions at their drug-binding interfaces are of particular translational interest. For four kinase–inhibitor pairs, we identified single substitutions at or near the drug-binding interface and quantified docking before and after substitution (Figure S16). In BRAF (a MAPK/ERK serine/threonine kinase; dabrafenib target^106,107^ ), K483R may weaken the interface by disrupting a salt bridge to the drug’s sulfonamide group^108^ (Figure 6H): Vina −11.2 → – 10.6 (∼0.6 kcal/mol). In EGFR (a receptor tyrosine kinase activated in LUAD/LSCC; target of the irreversible inhibitor afatinib, which covalently binds C797 ^109,110^ ), H805L in the kinase domain may alter local electrostatics and van der Waals contacts and shift the pocket conformation^111^ (Figure 6I): −8.6 → – 7.9 (∼0.7 kcal/mol). In MAPK1 (terminal MAPK/ERK kinase; ravoxertinib target^112,113^ ), G37Y in the conserved phosphate-binding loop adds a bulky aromatic side chain that may sterically hinder docking^114^ (Figure 6J): −9.0 → – 8.4 (∼0.6 kcal/mol). In MAPK14 (stress-activated kinase; target of doramapimod, which stabilizes a DFG-out state^115,116^ ), I141F in the adjacent allosteric pocket adds steric bulk that may impair the van der Waals contacts needed for high-affinity binding^117^ (Figure 6K): −10.7 → – 10.2 (∼0.5 kcal/mol). Because each shift (∼0.5–0.7 kcal/mol) lies within the typical error range of blind docking, none alone establishes a functionally significant change in binding affinity; each is a working hypothesis of weakened inhibitor binding that requires targeted experimental confirmation.

In each instance, whether through steric hindrance, disruption of crucial electrostatic or hydrophobic interactions, or alteration of allosteric conformational dynamics, these substitutions are predicted to modestly reduce binding affinity and should be treated as hypotheses requiring experimental validation rather than as established escape mechanisms. These analyses provide a structural starting point for investigating drug escape at the inhibitor-binding interface.

Beyond drug-target proteins, we also examined candidate substitutions in MHC/HLA proteins, whose substitutions could in principle alter antigen presentation and contribute to immune escape.^118–123^ Because these loci are hyperpolymorphic and highly prone to germline allelic-variation confounding, we present that analysis (with the appropriate caveats and after removing allelic-variation calls) in Supplementary Note 8 (Figure S18, Supplementary Datas 6, 7, 8 and 12).

## Discussion

In this study, the five analyzed cancer types are representative and span different tissues, helping reveal pervasive AA substitution patterns across cancer. For the proteomic search, we implemented a pipeline that balances search breadth against identification accuracy. To the best of our knowledge, empowered by PIPI-C, this pipeline is the first one to cover all types of AA substitutions without the numerical limitation of modifications in each PSM. Although PIPI-C outperforms the other engines in the regime that matters here, peptides carrying multiple co-occurring AA substitutions, its search results are not guaranteed to be error-free. Therefore, we use Comet as a validation step after the PIPI-C search, balancing search range and accuracy.

We identify consistent AA substitution patterns across five cancer cohorts, with E > M representing the most prevalent substitution among all E > X substitutions, and P > V emerging as the most frequent substitution within P > X substitutions. Given that translation fidelity is a critical determinant of gene expression precision, and ribosomal errors during mRNA-to-protein translation occur stochastically at an estimated rate of 10^–4^,^124^ we hypothesize that the prevalence of E > M and P > V substitutions may stem from two interrelated factors: 1. Heightened metabolic reliance on methionine^125^ and valine^126,127^ in cancer cells, and 2. elevated expression of methionyl-tRNA^128^ and valyl-tRNA synthetase^129^ in tumors. The consistent association of these high-frequency substitutions with cancer suggests their potential as putative therapeutic targets.

We next investigated the protein-level regulatory effects of AA substitutions by quantitative proteomics. Comparison of significantly regulated substitutions with documented UniProt variants^31^ showed 13% overlap, supporting the biological relevance of our findings (Supplementary Data S3). Most regulated substitutions are absent from OncoDB,^52^ a database of DNA/mRNA mutation-derived substitutions (Supplementary Data S4); however, absence from a somatic-mutation database does not by itself establish a nongenomic origin, as it may reflect incomplete coverage or low-frequency variants below sequencing detection thresholds. We therefore assessed undetected low-frequency somatic mutations and RNA editing using the matched CPTAC genomic data. Of 13,399 identified substitutions, none coincided with a somatic missense mutation at the same position and amino-acid change in the matched patient, and 61% require two or more base changes and therefore cannot arise from a single somatic point mutation or a single RNA-editing event; these are inaccessible to conventional genomic/transcriptomic analysis and identifiable only by direct proteome-scale profiling (Figure S17, Supplementary Data S13). Recent proteome-scale evidence indicates that such substitutions can arise from alternate translation of the genetic code.^7^ The remaining 39% are single-nucleotide-accessible (with 3.2% and 2.9% codon-consistent with A-to-I and C-to-U editing), for which an undetected low-frequency DNA/RNA variant, including germline variation not covered by our somatic-only cross-check, cannot be excluded. We emphasize that our dual-search strategy makes these candidate substitutions identifiable at the proteome scale (Figure S17).

Our pipeline also provides a framework for generating hypotheses about drug resistance and candidate immune escape in cancer cells. AA substitutions are only one possible contributor to drug escape; an exclusive focus neglects alternative pathways such as epigenetic alterations, metabolic reprogramming, and upregulation of efflux pumps, which can confer escape independent of substitutions in the drug’s target protein.^130^ Similarly, the candidate MHC-binding analysis is predictive: reduced predicted MHC binding affinity in substitution-modified sequences does not by itself establish immune escape, which can also arise through mechanisms such as T cell receptor escape. These predictions serve as a preliminary filter, and candidate neoantigens require experimental validation to confirm their ability to elicit an immune response.^131^

In this study, we identify several cancer-associated proteins exhibiting multiple significantly regulated AA substitutions. While these proteins are biologically relevant to cancer progression, we acknowledge several important limitations. First, clinical sample accessibility and experimental constraints prevent immediate wet-lab validation of our findings. Second, the vast combinatorial space of possible AA substitutions, where any residue may be replaced by any other AA, exceeds current biological annotation in the literature, making causal interpretation challenging. In addition, we cannot presently determine whether these AA substitutions represent drivers of oncogenesis or secondary effects of tumor biology. Mass spectrometry also provides only a population-averaged snapshot rather than single-molecule resolution. Consequently, identified AA substitutions likely represent aggregate modifications distributed across multiple protein molecules rather than co-occurring on individual proteins. Our structural visualizations, therefore, depict cumulative substitution patterns and their composite structural impacts. In addition, AlphaFold3 structural predictions carry inherent uncertainty, especially for substituted sequences that are under-represented in the training data; accordingly, the pLDDT-selected models are approximate, and the docking-based changes in binding affinity should be regarded as hypothesis-generating rather than quantitative. Developing methods to resolve these substitutions at the single-molecule resolution remains an important challenge for the field, as does understanding the functional consequences of specific substitution combinations within individual protein molecules.

## Conclusion

AA substitutions can alter protein structure and function in normal and cancer cells, yet prior studies have examined only a restricted number of them, in part because efficient open-search identification tools were lacking. To fill this gap, we used PIPI-C^17^ to profile all AA substitution types across diverse cancers. A few identified substitutions are documented as DNA- or mRNA-derived in OncoDB,^52^ but the majority (61%) are accessible only at the protein level, requiring two or more base changes and thus identifiable only by proteome-scale profiling (Figure S17, Supplementary Data S13)—consistent with an origin at the translation stage, as recently evidenced for alternate RNA decoding in mammals.^7^ Rather than assigning an exact proportion, we emphasize that PIPI-C makes these candidates identifiable at proteome scale; proteomic and protein-structure studies further support several of them, lending confidence to the pipeline.

For the first time, this study systematically characterizes multiple AA substitutions in cancer-associated proteins with proteomic-level validation and structural analyses independent of genomic/transcriptomic data. Across the five cohorts, we identify two clinically relevant HBB substitutions (F43S and E91D) previously documented to affect hemoglobin stability and function, a P584T substitution in FLNA domain 4 whose hydrophobic-to-hydrophilic (P > T) transition may perturb local interactions, and an A175N-substitution in ALDOB predicted to alter energy metabolism and molecular stability. We further noted predicted structural changes and predicted reductions in drug-target and candidate MHC-peptide binding affinity, which may inform hypotheses about drug and immune escape. Some substitutions are literature-supported while others are novel and require further validation, underscoring the need for expanded AA substitutomics investigations to define their precise roles in oncogenic processes.

Furthermore, the robustness of the AA substitutomics is validated. The substitutions exhibit strong reproducibility across two independent lung-cancer cohorts (LUAD and LSCC) sourced from distinct patient groups and TMT batches, which supports their reliability. For the cases we examined, literature integration showed concordance with independent studies, supporting the biological relevance of these substitutions. This study establishes the first AA substitutomics landscape, highlighting AA substitutions as candidate diagnostic markers that require dedicated clinical validation, while providing a foundation for future mechanistic investigations in cancer biology.

## Supplementary Material





























## Data Availability

The processed AA-substitutomics search results generated in this study are deposited on Zenodo and are freely available: the five CPTAC cohorts (10.5281/zenodo.13383150), the drug-target proteins (10.5281/zenodo.17669590), and the MHC proteins (10.5281/zenodo.17669609). The primary mass spectrometry data sets reanalyzed in this study are all publicly available: the five CPTAC proteomic cohorts from the CPTAC Data Portal/Proteomic Data Commons (PDC000204 for GBM, PDC000221 for HNSCC, PDC000153 for LUAD, PDC000234 for LSCC, and PDC000127 for ccRCC); the drug-target–enriched data sets from ProteomeXchange (PXD000788, PXD016465, PXD016505, and PXD016463); and the immunopeptidome (MHC) data sets from ProteomeXchange (PXD003790 and PXD000394). All accession numbers and repository links are also listed in the Materials and Methods. The PIPI-C for AA-substitution identification is publicly available at https://github.com/lsz1994024/PIPI-C.

## Notes

Combinatorial-optimization workflow of PIPI-C (Figure S1); false-positive control on the simulated data sets (Figure S2); false-positive control on the synthetic-peptide data sets (Figure S3); sensitivity and residue-level accuracy of PIPI-C, Open-pFind, MODplus, and MSFragger (Figure S4); identification of multimodification peptides (Figure S5); mistranslation-associated substitutions (Figure S6); site- and peptidoform-level validation of the identified substitutions (Figure S7); search results of the AA substitutomics pipeline (Figure S8); differential regulation of UPSPs carrying all modification types (Figure S9); histogram of the number of AA substitutions per protein, before and after length normalization (Figure S10); abundance of proteins carrying multiple regulated substitutions (Figure S11); GO cellular-component enrichment (Figure S12); manually annotated MS/MS spectra of representative substitutions (Figure S13); GO molecular-function enrichment of down-regulated UPSPs (Figure S14); abundance of the drug-target proteins (Figure S15); Vina-score changes upon substitution for the drug-target pairs (Figure S16); single-nucleotide accessibility and genomic detectability of the identified substitutions (Figure S17); candidate AA substitutions in MHC/HLA proteins (Figure S18); parameters for the simulated data sets (Table S1); modifications used to generate the synthetic-peptide data sets (Table S2); parameters used in AlphaPeptDeep (Table S3); and ambiguous substitution patterns excluded from analysis (Table S4); implementation and performance of PIPI-C (Supplementary Note 1); experimental details of the false-positive control test (Supplementary Note 2); experimental details of the substitution-identification sensitivity test (Supplementary Note 3); experimental details of the multimodification detection test (Supplementary Note 4); PIPI-C results on mistranslating-tRNA mutant versus wild-type data (Supplementary Note 5); supplementary figures and tables for the main analyses (Supplementary Note 6); detailed per-protein AA-substitution descriptions (Supplementary Note 7); candidate AA substitutions in MHC/HLA proteins (Supplementary Note 8) (PDF)
Normalized ratios of each AA substitution type across the five cohorts (Supplementary Data S1) (XLSX)
Quantification results for the five cohorts (Supplementary Data S2) (XLSX)
AA substitution details of all proteins recorded in UniProt (Supplementary Data S3) (XLSX)
AA substitution details of the eight proteins (Supplementary Data S4) (XLSX)
AA substitution details of the drug-target proteins (Supplementary Data S5) (XLSX)
NetMHCpan 4.2 output for PLEC and LDHB (Supplementary Data S6) (XLSX)
AA substitution details of the MHC proteins (Supplementary Data S7) (XLSX)
NetMHCpan 4.2 predicted binders (Supplementary Data S8) (XLSX)
Benchmark of the four methods on the simulated datasets (Supplementary Data S9) (XLSX)
Site- and peptidoform-level validation results (Supplementary Data S10) (XLSX)
One-sample t-test, limma t-test, and mixed-effects model comparison (Supplementary Data S11) (XLSX)
Cross-check of annotated AA substitution against germline HLA alleles (Supplementary Data S12) (XLSX)
Cross-reference of AA substitutions against CPTAC somatic mutations (Supplementary Data S13) (XLSX)
