# Supplementary material for: Amino Acid Substitutomics: Profiling Amino Acid Substitutions at the Proteomic Scale Unveils Biological Implications and Escape Hypotheses in Cancer
Source: J Proteome Res. 2026 Aug 27;25(10):5452–68. doi: 10.1021/acs.jproteome.6c00455 (PMC13640512; doi:10.1021/acs.jproteome.6c00455)
Supplement: Supplementary file 1 [file pr6c00455_si_001.pdf]

Supplementary Information

for

Amino acid substitutomics: profiling amino acid substitutions at the proteomic scale unveils biological implications and escape hypotheses in cancer

Peize Zhao[1,†], Shengzhi Lai[2,†], Shuaijian Dai[4,†], Chen Zhou[2], Ning Li[3,*], and Weichuan Yu[1,2,*]

[1] Individualized Interdisciplinary Program,
The Hong Kong University of Science and Technology, Hong Kong, 999077, China
[2] Department of Electronic and Computer Engineering,
The Hong Kong University of Science and Technology, Hong Kong, 999077, China
[3] Division of Life Science,
The Hong Kong University of Science and Technology, Hong Kong, 999077, China
[4] Department of Biomedical Engineering, School of Basic Medical Sciences,
Central South University, Hunan Province, 410013, China
[†] The authors contribute equally to this paper
[*] Correspondence authors: W.Y. (eeyu@ust.hk) and N.L. (boningli@ust.hk)

Contents

Contents S2

1 Supplementary Note 1: Implementation and performance of PIPI-C S5

Figure S1. Schematic workflow for generating protein candidates. S9

2 Supplementary Note 2: The experimental details of the false positive control test S9

Table S1. Parameters used in AlphaPeptDeep for the simulated datasets in the false positive control test. S10

Figure S2. Results of PIPI-C, Open-pFind, MODplus and MSFragger on the simulation datasets without AA substitutions. S11

Table S2. Information of the synthetic datasets in false positive control test. S12

Figure S3. Results of four open-search engines on the synthetic peptide datasets without AA substitutions. S13

3 Supplementary Note 3: The experimental details of the AA substitutions identification sensitivity test. S14

Table S3. Parameters used in AlphaPeptDeep for the simulated datasets in the test for sensitivity of detecting AA substitutions. S15

Figure S4. Sensitivity and accuracy of four open-search engines on simulated datasets containing 1–3 AA substitutions per peptide. S18

4 Supplementary Note 4: The experimental details of the test for detecting multiple modifications. S18

Figure S5. Number of PSMs with different numbers of enriched PTMs in one peptide. . S19

5 Supplementary Note 5: PIPI-C search result on proteomic data of mistranslating tRNA mutant and wild type. S20

Figure S6. Boxplot showing the numbers of PIPI-C identified AA substitutions are significantly different between the mutant datasets and the WT datasets. . . S21

6 Supplementary Note 6: Supplementary figures and tables for the main analyses S22

Figure S7. Site- and peptidoform-level support for the identified AA substitutions. . . . S22

Table S4. The ambiguous AA substitutions have the same mass shift as other modifications. . . . S23

Figure S8. The search results of the AA substitutomic pipeline. . . . S24

Figure S9. Volcano plots of quantification results based on the validated PSMs with modifications from five cohorts. . . . S25

Figure S10. The histogram of the number of identified AA substitutions in proteins. . . . S25

Figure S11. The abundance of the proteins that have multiple significantly regulated AA substitutions S25

Figure S12. The GO cellular component enrichment results based on the significantly regulated UPSPs. . . . S26

Figure S13. Manually annotated MS/MS spectra supporting eight representative AA substitutions. . . . S27

Figure S14. The GO molecular function enrichment results based on the down-regulated UPSPs. . . . S28

Figure S15. The abundance of the proteins that have multiple AA substitutions and the cancer drug target proteins from the PIPI-C's identification results. . . . S29

Figure S16. The change of Vina score change before and after the AA substitutions of the four drug-target pairs. . . . S29

Figure S17. Most identified AA substitutions cannot be explained by matched somatic mutations or by single canonical RNA-editing events. . . . S30

7 Supplementary Note 7: Detailed per-protein AA-substitution descriptions S31

7.1 Extracellular exosome proteins S31

7.2 Actin filament binding proteins S34

7.3 Glycolysis and gluconeogenesis proteins S36

8 Supplementary Note 8: Candidate AA substitutions in MHC/HLA proteins S38

Figure S18. AA substitutomics highlights candidate substitutions in MHC/HLA proteins S41

9 Supplementary Data Files **S42**

Supplementary Data S1. Normalized ratios of each AA substitution type across the five cohorts (xlsx) S42

Supplementary Data S2. Quantification results for the five cohorts (xlsx) S42

Supplementary Data S3. AA substitution details of all proteins recorded in UniProt (xlsx) S42

Supplementary Data S4. AA substitution details of the eight proteins (xlsx) S42

Supplementary Data S5. AA substitution details of the drug-target proteins (xlsx) S42

Supplementary Data S6. NetMHCpan 4.2 output for PLEC and LDHB (xlsx) S42

Supplementary Data S7. AA substitution details of the MHC proteins (xlsx) S42

Supplementary Data S8. NetMHCpan 4.2 predicted binders (xlsx) S42

Supplementary Data S9. Benchmark of the four methods on the simulated datasets (xlsx) S42

Supplementary Data S10. Site- and peptidoform-level validation results (xlsx) S42

Supplementary Data S11. One-sample t-test, limma t-test, and mixed-effects model comparison (xlsx) S42

Supplementary Data S12. Cross-check of annotated AA substitution against germline HLA alleles (xlsx) S42

Supplementary Data S13. Cross-reference of AA substitutions against CPTAC somatic mutations (xlsx) S42

1 Supplementary Note 1: Implementation and performance of PIPI-C

PIPI-C[1] formulates an optimization framework that addresses the challenge of identifying peptides with multiple PTMs from a combinatorial perspective, building upon the foundational concepts established by PIPI[2] and PIPI2.[3] In the identification process, PIPI-C extracts short AA segments as tags from the MS2 spectra based on the relevant peaks. These tags are then employed to retrieve candidate sequences from an FM-indexed protein database[4] using a fuzzy and bidirectional matching technique. The mass difference between the peptide sequence and the precursor ion of the MS2 spectrum is interpreted as the total mass of potential PTMs. This information is utilized to construct mixed integer linear programming (MILP) models, which can be solved using Gurobi.[5] Subsequently, peptide candidates with characterized PTM patterns are assembled and reranked through a protein feedback module,[6] allowing PIPI-C to generate a PSM list while controlling the FDR using a target-decoy strategy.

For tag extraction, PIPI-C combines tags of varying lengths to enhance the quality of the candidate list, leveraging the FM-index, which facilitates efficient querying of sequences of any length within the protein database. In the matching process, all target and decoy protein sequences are concatenated into a single long sequence to create the original FM-indexed database (o-FMDB). However, it is important to note that even a single erroneous AA in a tag can lead to retrieval failures. To address this, a fuzzy matching strategy is employed, which records the matched AAs and outputs the longest successfully matched substring. Nevertheless, fuzzy matching alone is inadequate when erroneous AAs are located at the beginning of a tag, as this results in initial matching errors. To overcome this limitation, a bidirectional matching approach is utilized alongside an additional reversed FM-indexed database (r-FMDB) generated from the reversed long sequence. In this setup, we attempt to match the reversed tag against the r-FMDB, where the backward matching of the reversed tag corresponds to a forward matching of the original tag. By integrating this fuzzy and bidirectional matching strategy, we consistently capture the correct portion of a tag, regardless of whether the erroneous AA is positioned at the N-terminus or C-terminus, thereby enhancing

candidate retrieval sensitivity. Subsequently, overlapping tags are merged into a single entity if their peaks align, while tags with lower intensity are discarded as noise. Finally, peptide backbone candidates are ranked based on the cumulative intensities of the identified tags. This aggregation of evidence from multiple tags supporting the same peptide backbone sequence effectively minimizes redundant evaluations of individual tags.

Given a candidate pair consisting of a peptide backbone sequence and associated tags, the protein sequence can be divided into three sections: a N-terminal section, a C-terminal section, and a gap section defined by the tags. The mass shift within each section is determined based on the peak locations of the tags, calculated as the mass difference between the starting peak and the ending peak of adjacent tags. This indicates that the PTM patterns within these sections operate independently. Consequently, PIPI-C formulates the characterization of PTMs as an optimization problem by constructing an MILP model for each potential combination of PTMs (**Figure S1**). The objective of this model is to maximize the total intensity of the matched experimental peaks corresponding to all b- and y-ions. PIPI-C employs Gurobi to solve this optimization problem, ultimately yielding the optimized results for PTM characterization.

In addition to methodological advancements, PIPI-C has demonstrated high performance, surpassing two leading open-search methods, Open-pFind[7] and MODplus,[8] in a series of validation experiments. To assess the precision and sensitivity of backbone identification and PTM characterization, seven simulated datasets with varying signal-to-noise ratios (SNRs), generated by AlphaPeptDeep,[9] were utilized. Each MS2 spectrum predicted the intensities of b and y-ions using the protein Packet_Kmod as a template, sourced from ProteomeTools[10] through the ProteomeXchange Consortium[11] (data identifier PXD009449). Noise peaks were randomly added to the predicted signal peaks to produce complete MS2 spectra. Each dataset contained 124,248 MS2 spectra encompassing up to four PTMs from 18 selected modifications, distributed as follows: 135 spectra with 0 PTMs, 20,004 with 1 PTM, 92,357 with 2 PTMs, 11,263 with 3 PTMs, and 489 with 4 PTMs. Results indicated that as SNR decreased, both PIPI-C and Open-pFind maintained relatively stable performance, whereas MODplus exhibited a more rapid decline in effectiveness. PIPI-C particularly excelled with peptides featuring more than two PTMs. This performance was

further validated using 21 synthetic datasets,[10] which collectively included 1,023,540 MS2 spectra across 21 raw files, each synthesized with a maximum of one selected PTM on the specified AA. Despite Open-pFind's approach of characterizing PTMs through enumerating single PTM and 2-PTM combinations, PIPI-C consistently outperformed both Open-pFind and MODplus in these datasets. Moreover, PIPI-C's performance was validated using dimethyl-labeled soybean datasets, where peptides were labeled with either light or heavy dimethyl, ensuring no peptide contained both labels. This dataset comprised approximately 800,000 MS2 spectra from the ProteomeXchange Consortium (data identifier PXD034796). The performance evaluation relied on the number of PSMs with peptides fully labeled by either type of dimethyl label, revealing that PIPI-C identified 75% and 17% more fully labeled peptides than Open-pFind and MODplus, respectively. Additionally, performance on PTM combination identification was confirmed using the Petunia datasets (ProteomeXchange Consortium, data identifier PXD005470). In this search setting, enriched ubiquitination on lysine was treated as an unknown PTM, serving as a benchmark for identifying reliable PTM combinations. PIPI-C yielded more PSMs with PTM combinations, demonstrating superior capability in identifying multiple PTMs in real data. All validation results were filtered at a peptide-level FDR of 0.01, reinforcing that PIPI-C outperforms state-of-the-art open-search methods. PIPI-C is developed in Java, and its source code is publicly available on GitHub at `https://github.com/lsz1994024/PIPI-C`. All parameter files, result files, and simulation datasets are available on Zenodo at `https://doi.org/10.5281/zenodo.14885715`.

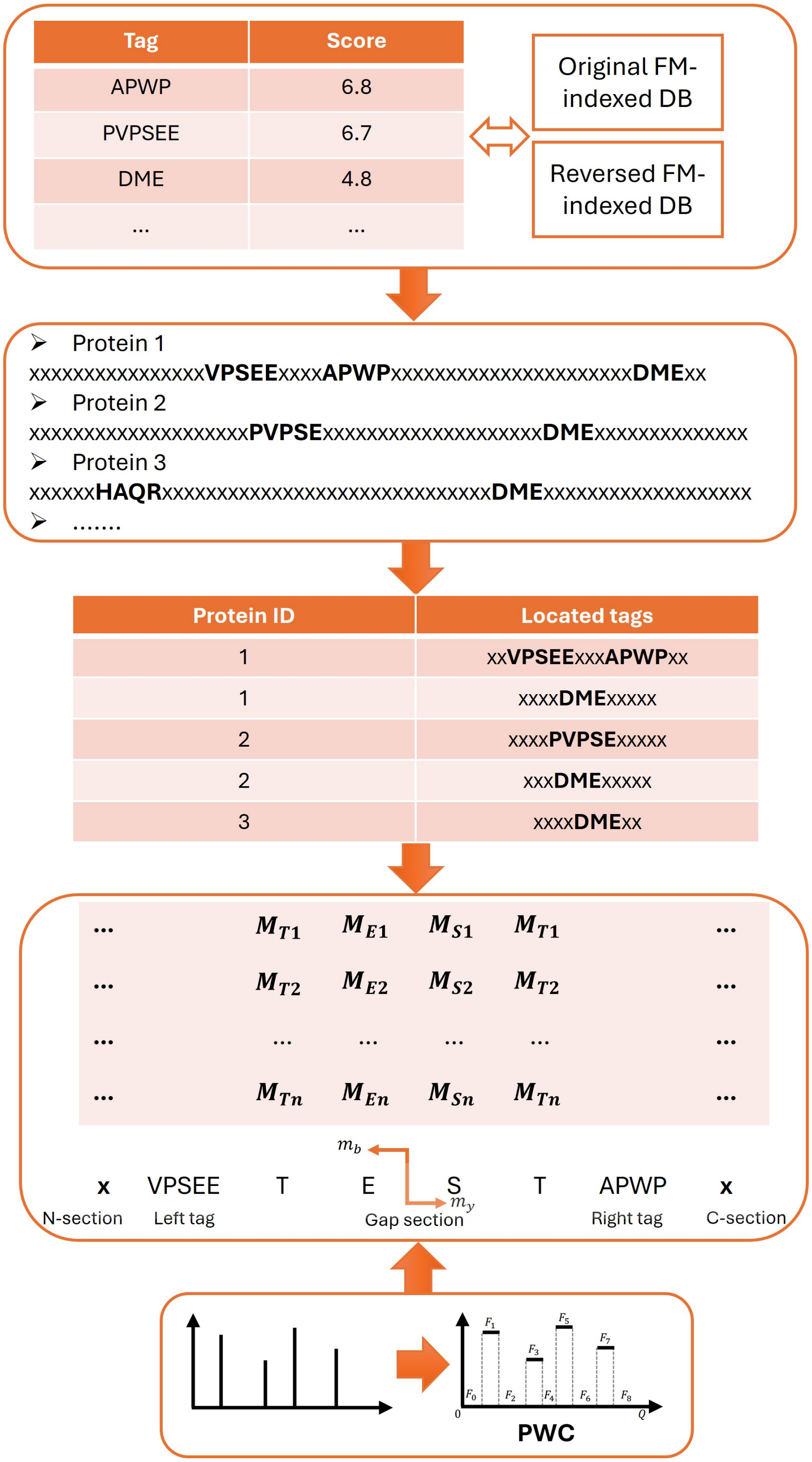

Tag
Score
APWP
6.8
PVPSEE
6.7
DME
4.8
...
...
Original FM-indexed DB
Reversed FM-indexed DB
Protein 1
xxxxxxxxxxxxxxxxVPSEExxxxAPWPxxxxxxxxxxxxxxxxxxxxxxxDMExx
Protein 2
xxxxxxxxxxxxxxxxxxxxPVPSExxxxxxxxxxxxxxxxxxxxDMExxxxxxxxxxxxxxx
Protein 3
xxxxxxHAQRxxxxxxxxxxxxxxxxxxxxxxxxxxxxxxDMExxxxxxxxxxxxxxxxxxx
....
Protein ID
Located tags
1
xxVPSEExxxAPWPxx
1
xxxxDMExxxxx
2
xxxxPVPSExxxxx
2
xxxDMExxxxx
3
xxxxDMExx
M_{T1} M_{E1} M_{S1} M_{T1}
M_{T2} M_{E2} M_{S2} M_{T2}
M_{Tn} M_{En} M_{Sn} M_{Tn}
m_b
m_y
x
VPSEE
T
E
S
T
APWP
x
N-section
Left tag
Gap section
Right tag
C-section
PWC

Figure S1: Schematic workflow for generating protein candidates. Initially, extracted tags are ranked based on their scores and used to retrieve protein candidates from FM-indexed databases. Next, the tags are aligned with the retrieved protein sequences using a fuzzy and bidirectional matching strategy. Subsequently, the protein sequences that contain the mapped tags are regarded as candidates and ranked according to the cumulative scores of the identified tags. Following this, the peaks of an MS2 spectrum are depicted as a piece-wise constant function. Finally, in both the N-terminal and C-terminal sections, amino acids are classified into fixed, optional, and infeasible categories to support the construction of the MILP model.

2 Supplementary Note 2: The experimental details of the false positive control test

In this study, we aim to profile various amino acid (AA) substitutions across five cancer cohorts. We design a series of experiments to evaluate and select the most suitable open search method for our research needs. The four candidate methods are PIPI-C,[1] Open-pFind (version 3.2.0),[7] MODplus (version 2.01),[8] and MSFragger (version 4.2).[12]

The first experiment is a false positive control test with simulation datasets. In this paper, we use AlphaPeptDeep[9] to generate simulation datasets. In each simulated MS2 spectrum, the intensities of b-ions and y-ions are predicted, and the noise peaks are randomly incorporated into the predicted peaks to produce the final MS2 spectra. In the false positive control test, we generate three simulation datasets with the average SNRs as 0.5, 1.0, and 2.0. The signal-to-noise ratio (SNR) is defined as:

$$\mathrm{SNR} = \sum I_s^2 / \sum I_n^2. \tag{S1}$$

Where I_s means the intensity of the signal peak and I_n means the intensity of the noise peak. Each of the simulation datasets used for the false positive control test has 30,000 spectra. The parameters for AlphaPeptDeep to generate the simulation datasets in false positive control test are detailed in **Table S1**. The four methods are used with the following parameters: the MS1 tolerance: 10 ppm; the MS2 tolerance: 0.01 Da; the max cleavage site: 2; the enzyme: trypsin; the

peptide length: from 6 to 25; the fixed modification: carbamidomethyl on cysteine; the variable modifications: the UNIMOD[13] database including all types of AA substitutions and common post-translational modifications (PTMs); modifications search range: ±250 Da. The decoy proteins are added for FDR control with the target-decoy strategy. And the delta-mass localization function is enabled for MSFragger. The rest of the settings are kept as the default. The human protein database is downloaded from UniProt[14] (January 2024). These search parameters are used in all testing experiments unless otherwise informed. To evaluate the performance of the false positive control test, since there are no AA substitutions or any modifications in the simulation dataset, the lower the ratio of identified AA substitutions, the better the performance. This metric scores the rate at which substitutions are reported and does not test whether a reported substitution is assigned to the correct residue; residue-level accuracy is evaluated separately in Supplementary Note 3. The heatmaps show the proportion of all kinds of AA substitutions (**Figure S2**). The proportion of each AA substitution is normalized by the number of the original AAs in the result peptide spectra matches (PSMs):

$$\text{ratio} = \frac{\text{number of identified AA1>AA2 substitutions}}{\text{number of AA1 in the results}}. \tag{S2}$$

Table S1: Parameters used in AlphaPeptDeep for the simulated datasets in the false positive control test.

**Enzyme**	trypsin
**Instrument**	Lumos
**Precursor charge**	2-4
**Peptide length**	6-25
**Max missed cleavages**	2
**Fixed modification**	Carbamidomethyl@C
**Num variable modifications**	0

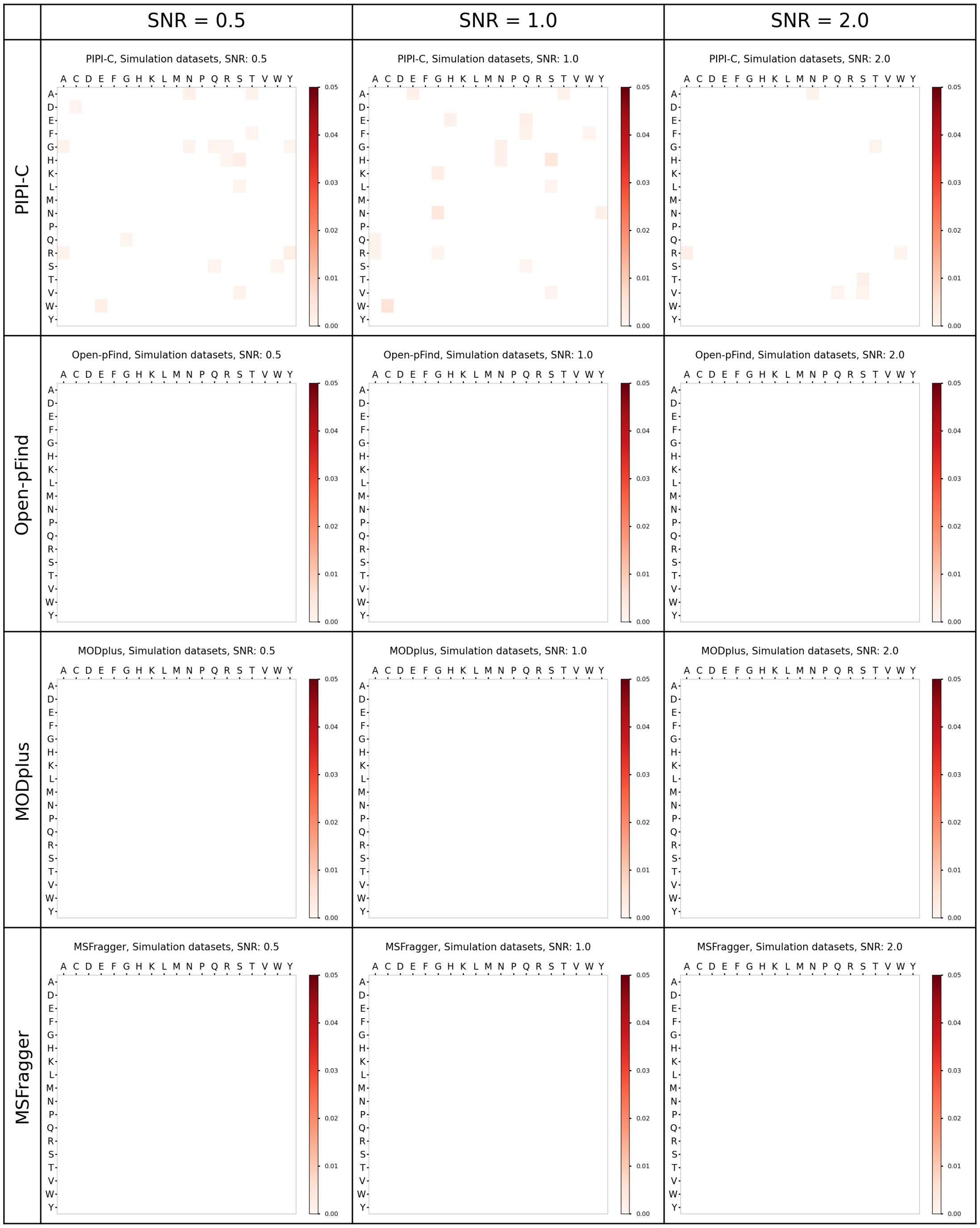

Figure S2: Results of PIPI-C, Open-pFind, MODplus and MSFragger on the simulation datasets without AA substitutions, at three signal-to-noise ratios (SNR = 0.5, 1.0 and 2.0). The spectra number of each dataset is 30,000. In each heat map the y-axis is the original AA and the x-axis is the substituting AA; the colour represents the false-identification ratio, defined as the number of identified AA1>AA2 substitutions divided by the number of AA1 residues in the identified PSMs. Because the datasets contain no AA substitutions, every identification shown is a false positive, and a lower ratio indicates better performance. All engines were filtered at 1% target-decoy FDR.

From **Figure S2**, we can find that Open-pFind and MODplus detect no AA substitutions, while PIPI-C achieves a maximum ratio of approximately only 0.5% when the SNR is set to 1.0. The results of the false positive control test using the simulated datasets indicate a comparable performance among the four methods.

To conduct a more stringent false positive control test, we utilize 21 synthetic datasets obtained from ProteomeTools,[10] which are sourced from the ProteomeXchange Consortium[11] with the data identifier PXD009449. The raw files are converted to the mgf format using ProteomeWizard MSConvert.[15] These datasets consist of 21 raw files containing a total of 1,023,540 MS2 spectra. In each raw file, the MS2 spectra are generated with at most one PTM on the specific AA. The details of the selected PTM and the modified residue are shown in **Table S2**. Given that there are no AA substitutions in the synthetic datasets, a lower ratio of identified AA substitutions indicates better performance. The normalized ratio of each type of AA substitution from the 21 datasets is illustrated in **Figure S3**.

Table S2: Information of the synthetic datasets in false positive control test.

ID	Amino acid	Modification name	Monoisotopic mass	Number of MS2
1	Lysine	Formylation	27.995	51861
2	Lysine	Acetylation	42.010	50511
3	Tyrosine	Phosphorylation	79.966	55131
4	Lysine	Methylation	14.016	49086
5	Lysine	Biotinylation	226.078	43572
6	Lysine	Butyrylation	70.042	48573
7	Lysine	Crotonylation	68.026	49398
8	Lysine	Dimethylation	28.031	47914
9	Lysine	Malonylation	86.000	50877
10	Lysine	Succinylation	100.016	49896
11	Proline	Hydroxyproline	15.995	46893
12	Lysine	Glutarylation	114.032	49197
13	Lysine	GlyGlycylation	114.043	47664
14	Lysine	Hydroxyisobutyrylation	86.037	49329
15	Lysine	Propionylation	56.026	49305
16	Lysine	Trimethylation	42.047	49296
17	Arginine	Citrullination	0.984	50087
18	Arginine	Dimethylation_asym	28.031	45183
19	Arginine	Dimethylation_symm	28.031	45666
20	Arginine	Methylation	14.016	45396
21	Tyrosine	Nitrotyrosine	44.985	48705

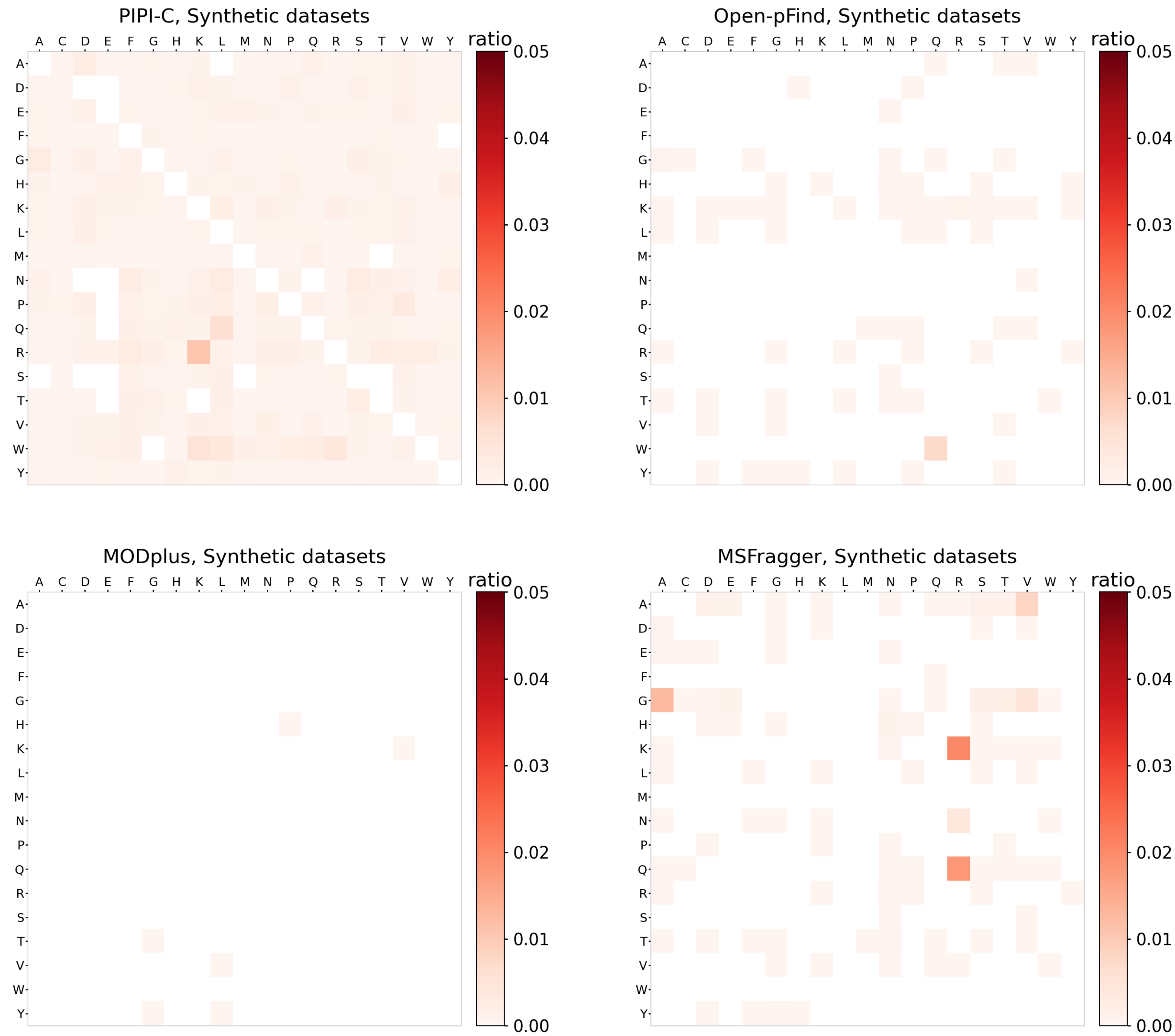

Figure S3: Results of PIPI-C, Open-pFind, MODplus and MSFragger on the 21 synthetic peptide datasets without AA substitutions. The PSM number of the synthetic dataset is 1,023,540.

During our experiments with the synthetic datasets, we find the two substitutions S>D and T>E were frequently identified. Upon investigating the modification mass shifts of these two AA substitutions, we notice that both masses are equal to 27.995, which is identical to the mass of Formylation. Consequently, these two AA substitutions could be wrongly associated with Formylation, potentially arising from sample preparation steps. In mass spectrometry-based methods, it is not possible to distinguish such mass-identical AA substitutions and PTMs. Therefore, we find 20 modification patterns that have the same modification mass shift with AA substitutions (**Table S4**) and exclude these AA substitutions from the cancer data analysis. Based on **Figure S3**, all four engines occasionally misassign the defined modification as an AA substitution, at comparable low rates: the maximum per-substitution false-identification ratio ranged from approximately 3.8% (PIPI-C) to 5.3% (MSFragger), and the mean per-substitution ratio from 2×10^{-4} (Open-pFind) to 8×10^{-4} (PIPI-C).

Based on the low identification rates discussed above, it can be concluded that the four methods exhibit comparable and satisfactory performance in the false positive control tests.

3 Supplementary Note 3: The experimental details of the AA substitutions identification sensitivity test.

In the test for sensitivity of detecting AA substitutions, we utilize AlphaPeptDeep to generate three simulation datasets with varying numbers of AA substitutions in a single peptide: one AA substitution per peptide (1-AASub dataset), two AA substitutions per peptide (2-AASub dataset), and three AA substitutions per peptide (3-AASub dataset). Given that carbamidomethyl is considered the fixed modification on cysteine and isoleucine and leucine are treated as leucine, there are a total of 324 types of AA substitutions in this study. Each simulation dataset consisted of 324 spectrum files, with each file containing 1000 spectra. Among these spectra, 5% include one type of AA substitution. The parameter configurations for AlphaPeptDeep are outlined in **Table S3**. The search parameters remain consistent with those employed in the false positive control test.

Table S3: Parameters used in AlphaPeptDeep for the simulated datasets in the test for sensitivity of detecting AA substitutions.

**Enzyme**	trypsin
**Instrument**	Lumos
**Num variable modifications**	1 or 2 or 3
**Max missed cleavages**	2
**Precursor charge**	2-4
**Peptide length**	6-25
**Fixed modification**	Carbamidomethyl@C
**Variable modification**	324 kinds of AA substitutions

Since the ratio of spectra containing AA substitutions is known to be 5%, we utilize the absolute difference between the identified AA substitution ratio and 5% as the evaluation metric. As in Supplementary Note 2, this metric evaluates the identified rate rather than the correctness of the residue assignment. Because the simulated datasets record the true substituted positions, we additionally re-scored all four engines at the peptidoform and residue levels against this ground truth. A smaller difference indicates that the AA substitutions ratio closely aligns with 5%, signifying better sensitivity in detecting AA substitutions. The outcomes of the four methods across the three simulation datasets with varying numbers of AA substitutions per peptide are depicted in **Figure S4A**. Upon analyzing the results of the sensitivity test, it is observed that Open-pFind exhibits slightly better performance in the 1-AASub simulated dataset than the other methods, and MSFragger performs comparably to PIPI-C in this setting. However, in scenarios involving multiple substitutions, PIPI-C surpasses the other three. Open-pFind and MSFragger only correctly identify a limited number of AA substitutions, falling short of meeting the desired criteria.

To establish whether this behaviour reflects a parameter choice or an intrinsic property of open searching, we examined the MSFragger publication and the FragPipe documentation. The literature search shows that MSFragger reports a single delta mass per peptide-spectrum match; when two or three modifications co-occur on one peptide, the observed shift is their sum, which corresponds to no individual modification and therefore cannot be decomposed into residue-level events. FragPipe's documentation states that a mass shift arising from modifications on two separate residues "cannot be localized well", and that multiple lower-case letters in the localization output denote ambiguous localization.

This limitation is architectural rather than parametric, and it is not specific to MSFragger: it applies to any method that summarises a peptide by a single mass offset. We therefore did not include MSFragger in the multi-modification identification test of Supplementary Note 4, in which several enriched PTMs occur on the same peptide, nor in the mistranslating-tRNA analysis of Supplementary Note 5, since in both settings a single delta mass cannot be resolved to the individual modified residues and the comparison would not be informative. MSFragger's performance is reported in full for the false-positive control (Supplementary Note 2) and the sensitivity and residue-level accuracy tests (this Note), where it is directly comparable with the other engines.

The metric above compares the rate of identified substitutions with the known 5%, but does not establish that a substitution was placed on the correct residue. Because the simulated datasets record the true substituted positions, we re-scored all four engines under a stricter criterion: a spectrum counts as correct at the peptidoform level only if the reported bare peptide sequence matches the true sequence exactly, and at the residue level only if every true substitution on that spectrum is localized to the exact position. As the results shown in the **Figure S4B**, for singly substituted peptides Open-pFind localizes more accurately. And the performance of engines separate when substitutions co-occur, and the margin widens with their number. PIPI-C's peptidoform-level and residue-level accuracy is about 1.4-fold over the next-best engine at two substitutions and about 2.1-fold at three substitutions scenario.

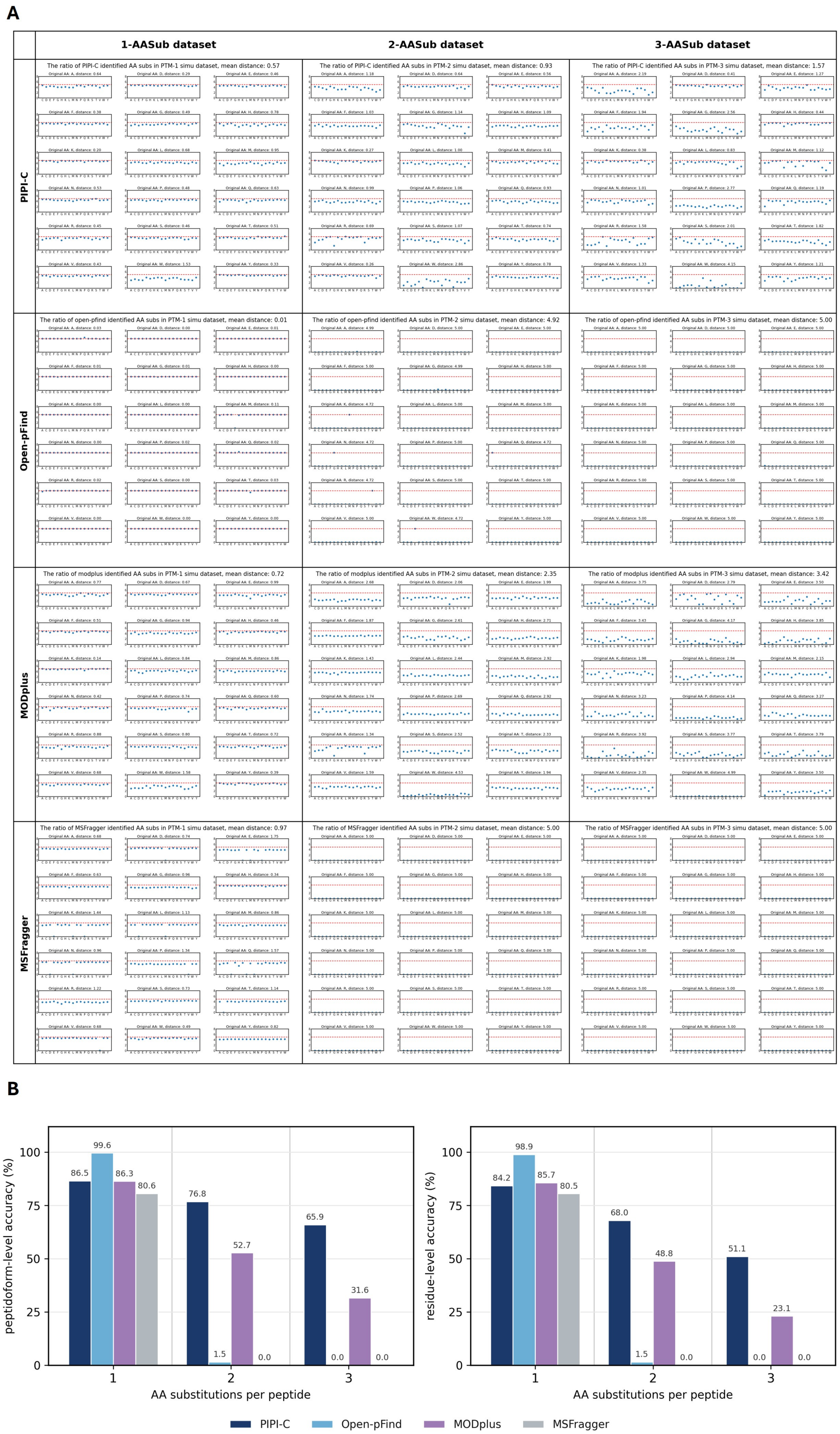
A
1-AASub dataset
2-AASub dataset
3-AASub dataset
PIPI-C
Open-pFind
MODplus
MSFragger
The ratio of PIPI-C identified AA subs in PTM-1 simu dataset, mean distance: 0.57
The ratio of PIPI-C identified AA subs in PTM-2 simu dataset, mean distance: 0.93
The ratio of PIPI-C identified AA subs in PTM-3 simu dataset, mean distance: 1.57
The ratio of open-pfind identified AA subs in PTM-1 simu dataset, mean distance: 0.01
The ratio of open-pfind identified AA subs in PTM-2 simu dataset, mean distance: 4.92
The ratio of open-pfind identified AA subs in PTM-3 simu dataset, mean distance: 5.00
The ratio of modplus identified AA subs in PTM-1 simu dataset, mean distance: 0.72
The ratio of modplus identified AA subs in PTM-2 simu dataset, mean distance: 2.35
The ratio of modplus identified AA subs in PTM-3 simu dataset, mean distance: 3.42
The ratio of MSFragger identified AA subs in PTM-1 simu dataset, mean distance: 0.97
The ratio of MSFragger identified AA subs in PTM-2 simu dataset, mean distance: 5.00
The ratio of MSFragger identified AA subs in PTM-3 simu dataset, mean distance: 5.00
B
peptidoform-level accuracy (%)
86.5
99.6
86.3
80.6
76.8
1.5
52.7
0.0
65.9
0.0
31.6
0.0
residue-level accuracy (%)
84.2
98.9
85.7
80.5
68.0
1.5
48.8
0.0
51.1
0.0
23.1
0.0
AA substitutions per peptide
PIPI-C
Open-pFind
MODplus
MSFragger

Figure S4: Sensitivity and accuracy of four open-search engines on simulated datasets containing 1–3 AA substitutions per peptide.
(A) Results of PIPI-C, Open-pFind, MODplus and MSFragger on the simulation datasets with 1–3 AA substitutions in each peptide. In each subplot, the x-axis is the substituting AA, the y-axis is the ratio. The original AA and the mean distance between the ratio and 5% are in the title of the subplots. The horizontal red dashed line is 5%. The spectra number of each dataset is 324,000.
(B) Peptidoform- and residue-level accuracy of the four engines against the ground truth of the same datasets, which record the true substituted positions. A spectrum counts as correct at the peptidoform level only if the reported bare peptide sequence matches the true sequence exactly (left), and at the residue level only if every true substitution on that spectrum is localized to the exact position (right); 48,600 substitution-bearing spectra in total. Per-engine and per-file results are provided in Supplementary File 9.

4 Supplementary Note 4: The experimental details of the test for detecting multiple modifications.

In this paper, we aim to analyze the landscape and profile of all types of AA substitutions. Therefore, the ability to identify multiple AA substitutions in one peptide is crucial in method evaluation. Since there is no proteomic dataset with enriched AA substitutions, to fulfill this requirement, we use a proteomic dataset with serial enrichment of phosphorylation, ubiquitination, and acetylation.[16] The dataset is downloaded from the MassIVE Repository with the data identifier MSV000078509. After converting to MGF format, the dataset contains 612,348 spectra.

In **Figure S5**, we show the number of PSMs with different numbers of enriched PTMs in one peptide. The results from the three methods indicate that the three methods are comparable in identifying a single modification within a peptide. Additionally, when it comes to detecting multiple modifications in one peptide, PIPI-C demonstrates superior performance with many more identification results over both competing methods, with MODplus ranking as the second-best option.

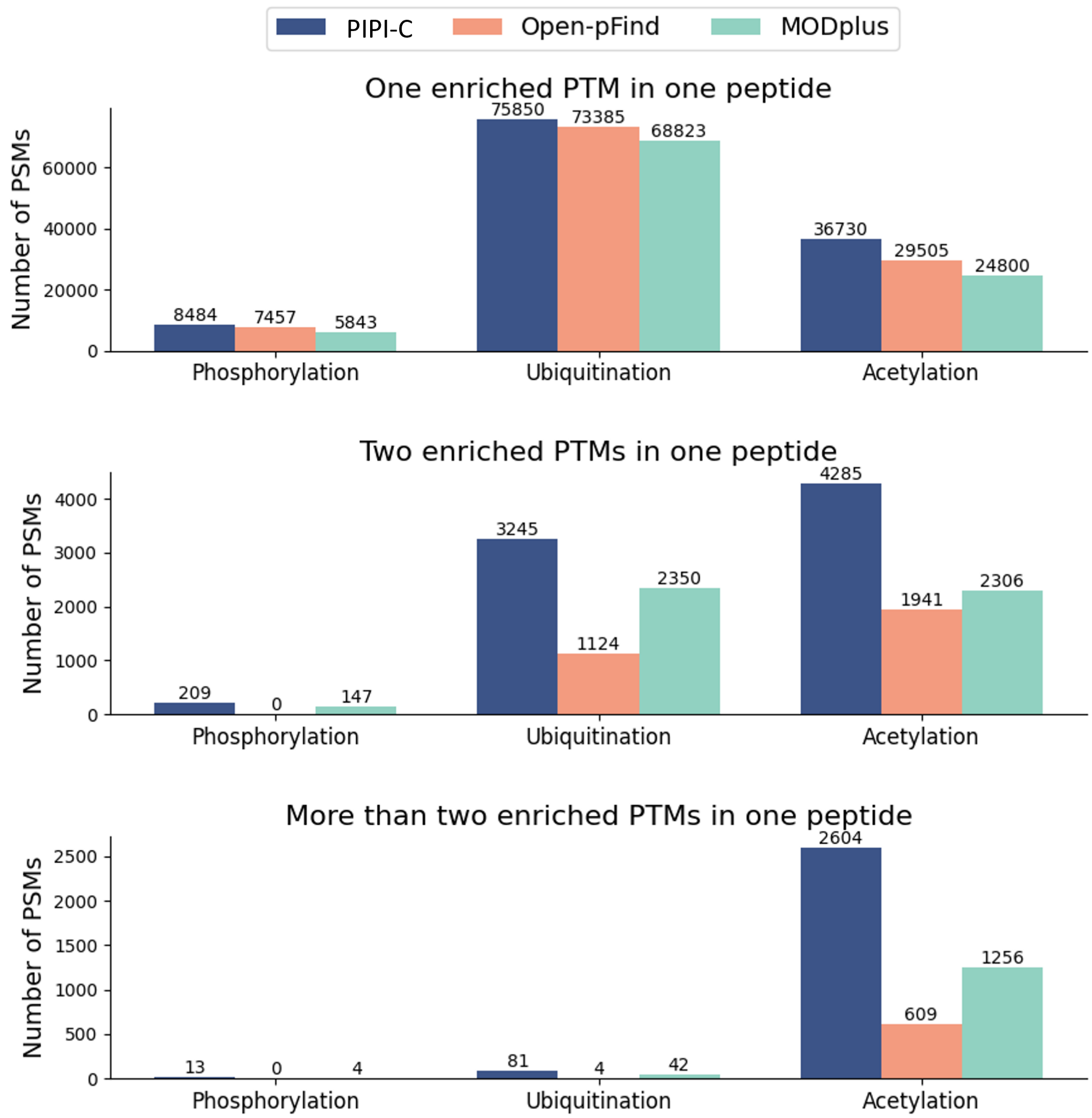

Figure S5: Number of PSMs with different numbers of enriched PTMs in one peptide from results of PIPI-C, Open-pFind, and MODplus on the serial PTM enrichment dataset. The dataset contains 612,348 spectra.

5 Supplementary Note 5: PIPI-C search result on proteomic data of mistranslating tRNA mutant and wild type.

In this section, we will showcase the power of PIPI-C in identifying AA substitutions on mistranslating tRNA mutant datasets, where the number of R>S substitution and P>A substitution is known to be more than in the wild-type (WT) dataset.[17] The dataset is downloaded from ProteomeXchange Consortium with the data identifier PXD025934 and the protein database of Saccharomyces cerevisiae (yeast) is downloaded from UniProt (October 2024). When growing yeast, two types of engineered tRNA are involved, therefore, the frequencies of R>S substitution and P>A substitution in the two mutant groups are higher than in the WT group

PIPI-C identifies 156,165 PSMs, 121,096 PSMs, and 152,371 PSMs for the WT group, R>S group, and P>A group, respectively. From **Figure S6**, we can find that PIPI-C successfully identifies more designed AA substitutions in each mutant group compared with the WT group and another mutant group. With this experiment, PIPI-C is proven to differentiate the mutant sample and the WT sample with different frequencies of AA substitutions. Such observation further encourages us to apply PIPI-C to the real cancer dataset and makes us more confident about PIPI-C's results.

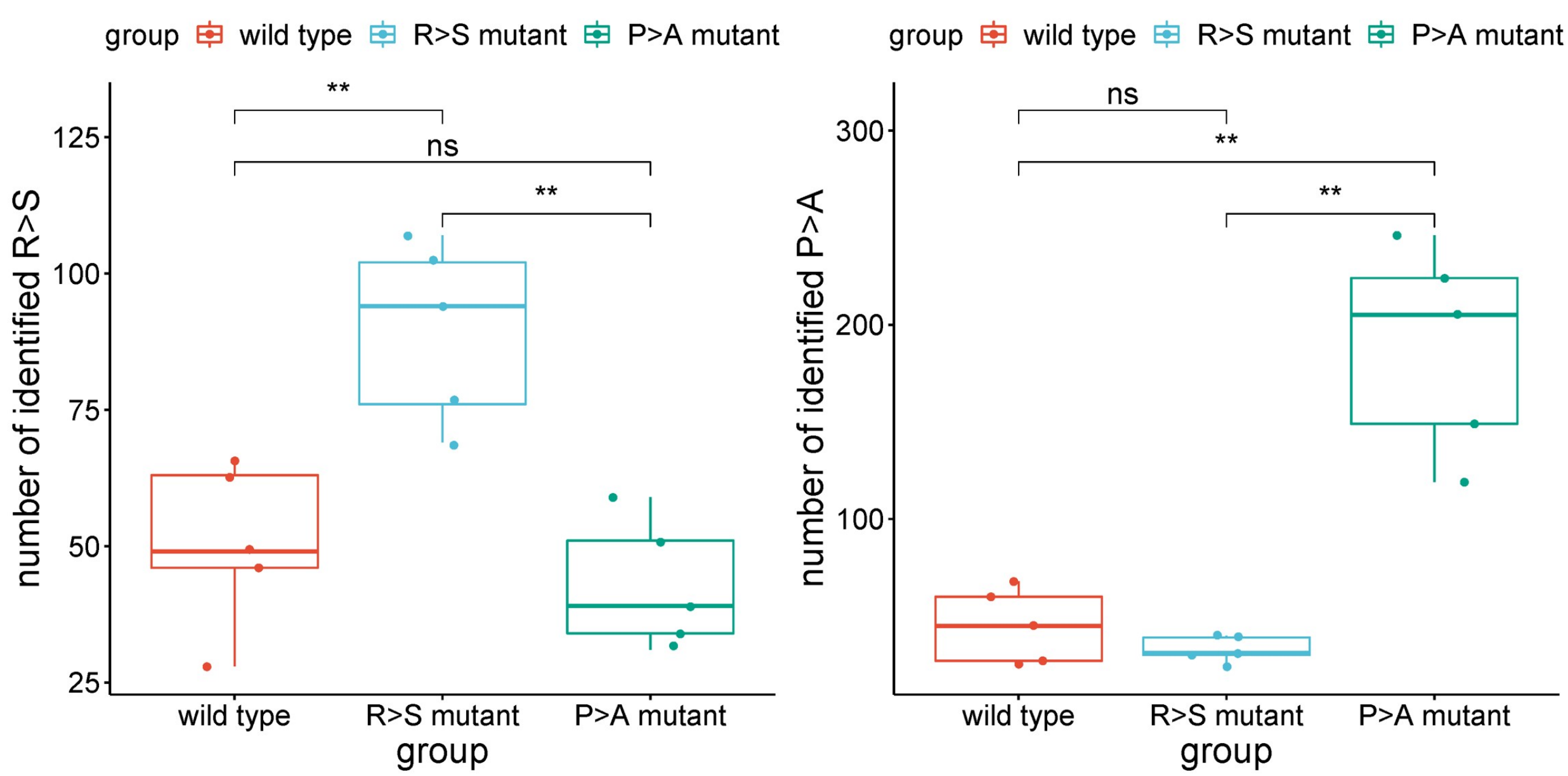

Figure S6: Boxplot showing the numbers of PIPI-C identified AA substitutions are significantly different between the mutant datasets and the WT datasets. Left: the number of identified R>S substitutions. Right: the number of identified P>A substitutions. T-test p-values are shown, **: p-value < 0.01, ns: p-value > 0.05.

6 Supplementary Note 6: Supplementary figures and tables for the main analyses

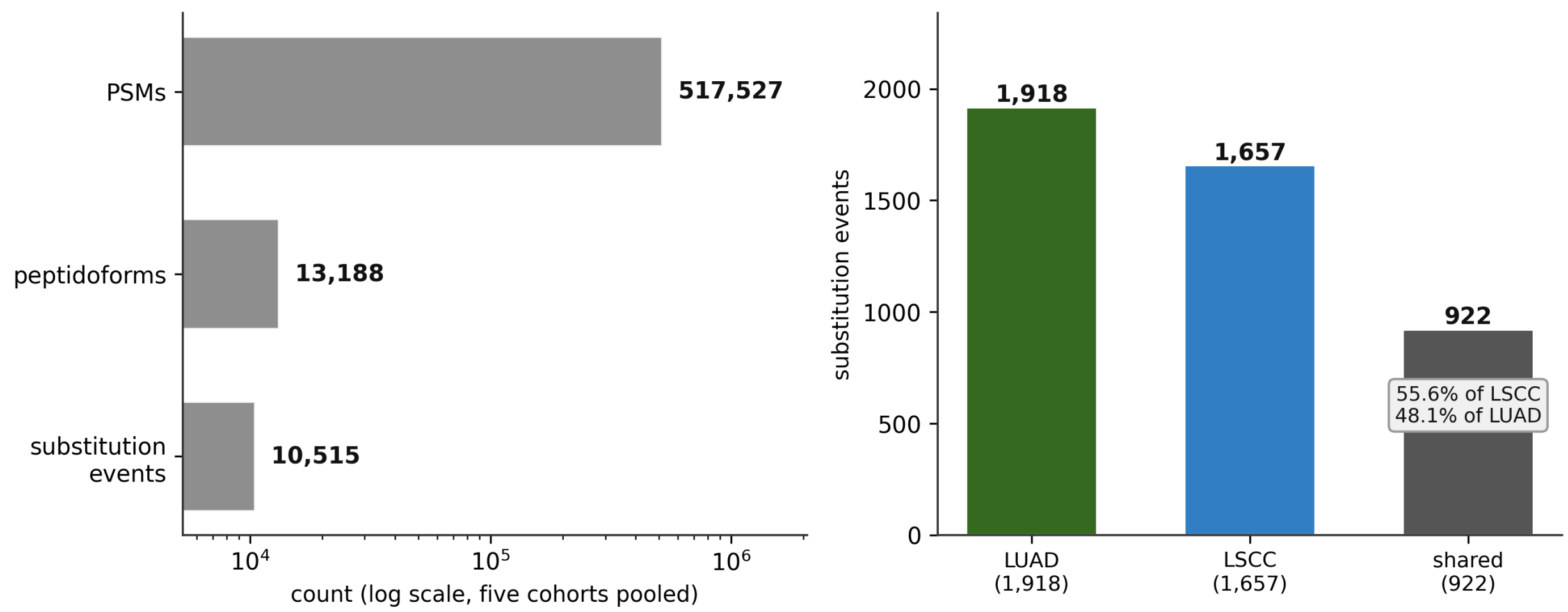

Figure S7: Site- and peptidoform-level support for the identified AA substitutions.
Left: Aggregation of substitution evidence across the five cohorts (counts pooled, log scale): 517,527 substitution peptide-spectrum matches (PSMs) collapse to 13,188 peptidoforms and 10,515 cohort-level substitution events. Right: Substitution events in the two lung-cancer cohorts, which derive from independent patient series and independent TMT batches: of 1,918 substitution events in LUAD and 1,657 in LSCC, 922 are identical in protein, residue and substitution, corresponding to 48.1% of LUAD and 55.6% of LSCC. Underlying per-cohort counts, the 922 shared substitution events and the check that no reported substitution is isobaric with a modification annotated on its own residue are provided in Supplementary File 10.

Table S4: The ambiguous AA substitutions have the same mass shift as other modifications.

Modification site	Monoisotopic mass	AA substitution name	Modification name
Aspartic acid	14.016	Asp>Glu	Methylation
Phenylalanine	15.995	Phe>Tyr	Oxidation
Methionine	-29.993	Met>Thr	Met>Hse
Asparagine	0.984	Asn>Asp	Deamidated
Glutamine	0.984	Gln>Glu	Deamidated
Serine	-15.995	Ser>Ala	Deoxydation
Serine	14.016	Ser>Thr	Methylation
Threonine	27.047	Thr>Lys	Ethylamino
Alanine	42.047	Ala>Xle	Trimethylation
Asparagine	15.000	Asn>Glu	Methylation+Deamidated
Asparagine	14.016	Asn>Gln	Methylation
Proline	31.990	Pro>Glu	Dioxidation
Serine	27.995	Ser>Asp	Formylation
Serine	42.011	Ser>Glu	Acetylation
Serine	44.008	Ser>Met	Delta:H(4)C(2)O(-1)S(1)
Threonine	27.995	Thr>Glu	Formylation
N-term	14.016	Asp>Glu<br>Ser>Thr<br>Val>Xle<br>Asn>Gln<br>Gly>Ala	Methylation
C-term	14.016	Asp>Glu<br>Ser>Thr<br>Val>Xle<br>Asn>Gln<br>Gly>Ala	Methylation
N-term	27.995	Ser>Asp<br>Thr>Glu	Formylation
N-term	42.011	Ser>Glu	Acetylation

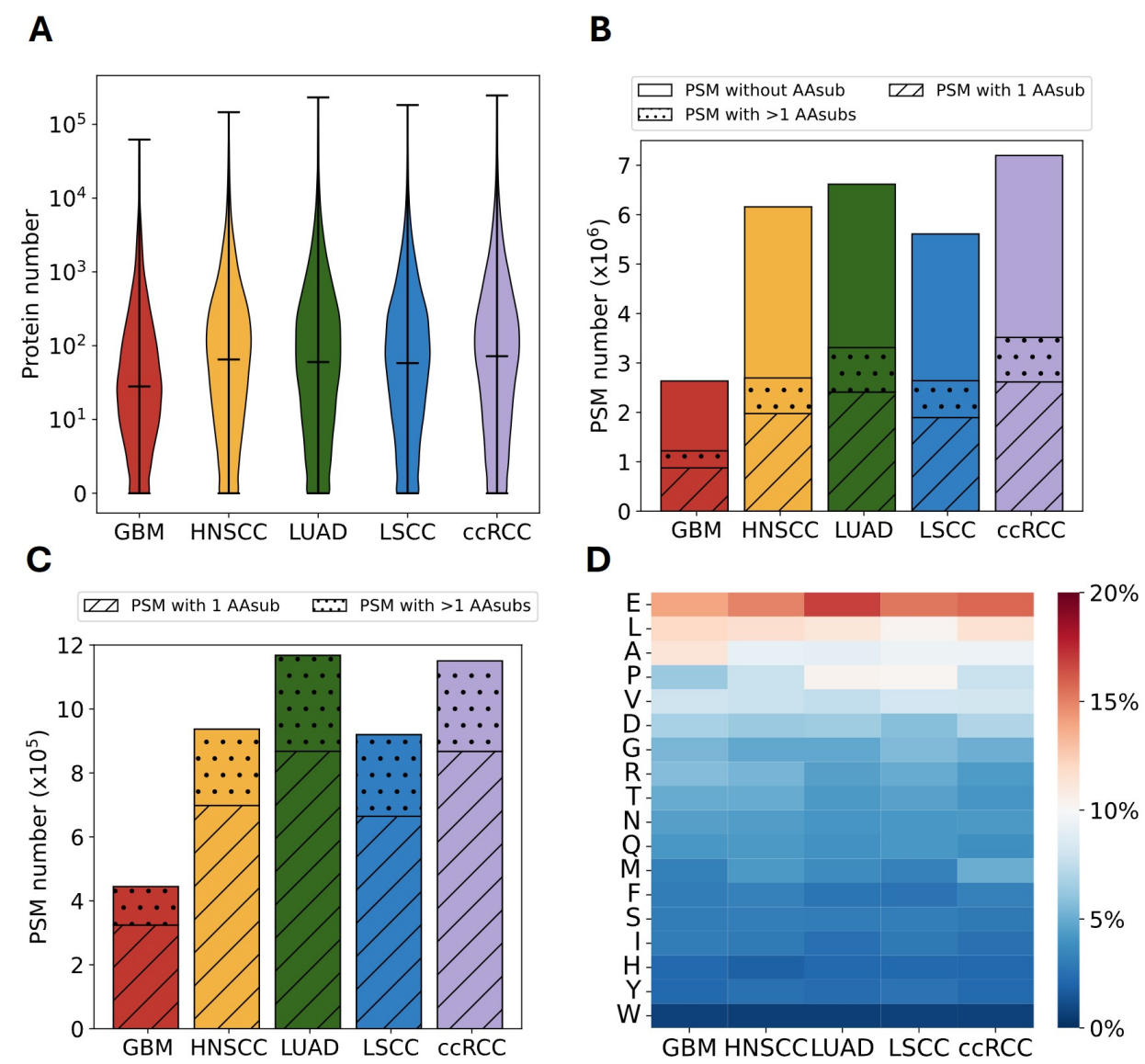

Figure S8: The search results of the AA substitutomic pipeline.

(A) Violin plot showing the PIPI-C-identified protein number of each cancer cohort. The cohorts are GBM (dark red), HNSCC (yellow), LUAD (green), LSCC (blue), and ccRCC (purple).

(B) The PSMs in PIPI-C's identification results. Clean section: PSMs without any AA substitutions. Slashed section: PSMs with one AA substitution. Dotted section: PSMs with more than one AA substitution. The cohorts are GBM (dark red), HNSCC (yellow), LUAD (green), LSCC (blue), and ccRCC (purple).

(C) Bar plot showing the number of PSMs in Comet's validated identification results. Slashed section: PSMs with one AA substitution. Dotted section: PSMs with more than one AA substitution. The cohorts are GBM (dark red), HNSCC (yellow), LUAD (green), LSCC (blue), and ccRCC (purple).

(D) Normalized ratio of substituted AAs in each cohort.

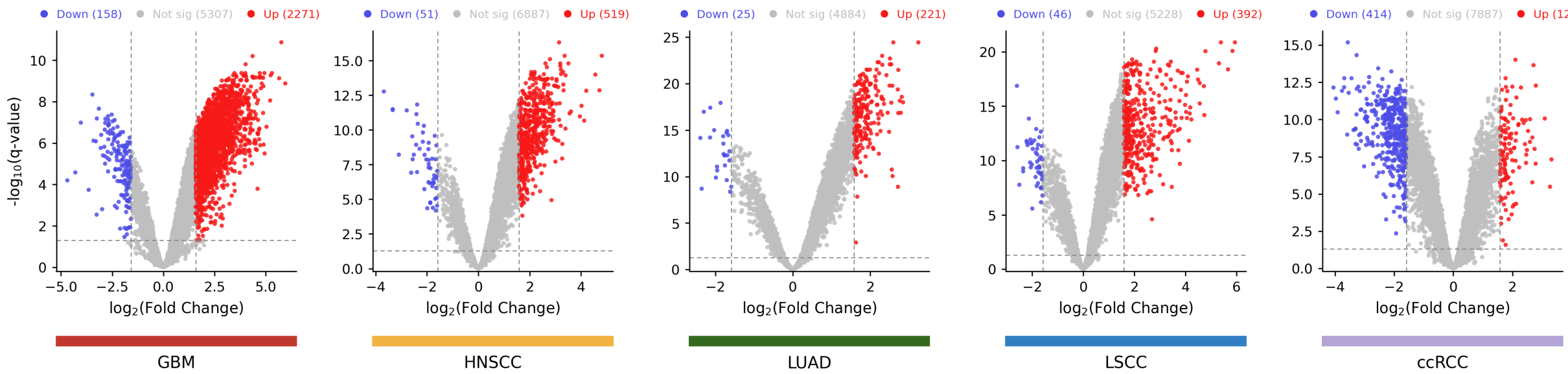

Figure S9: Volcano plots of quantification results based on the validated PSMs with modifications from five cohorts.

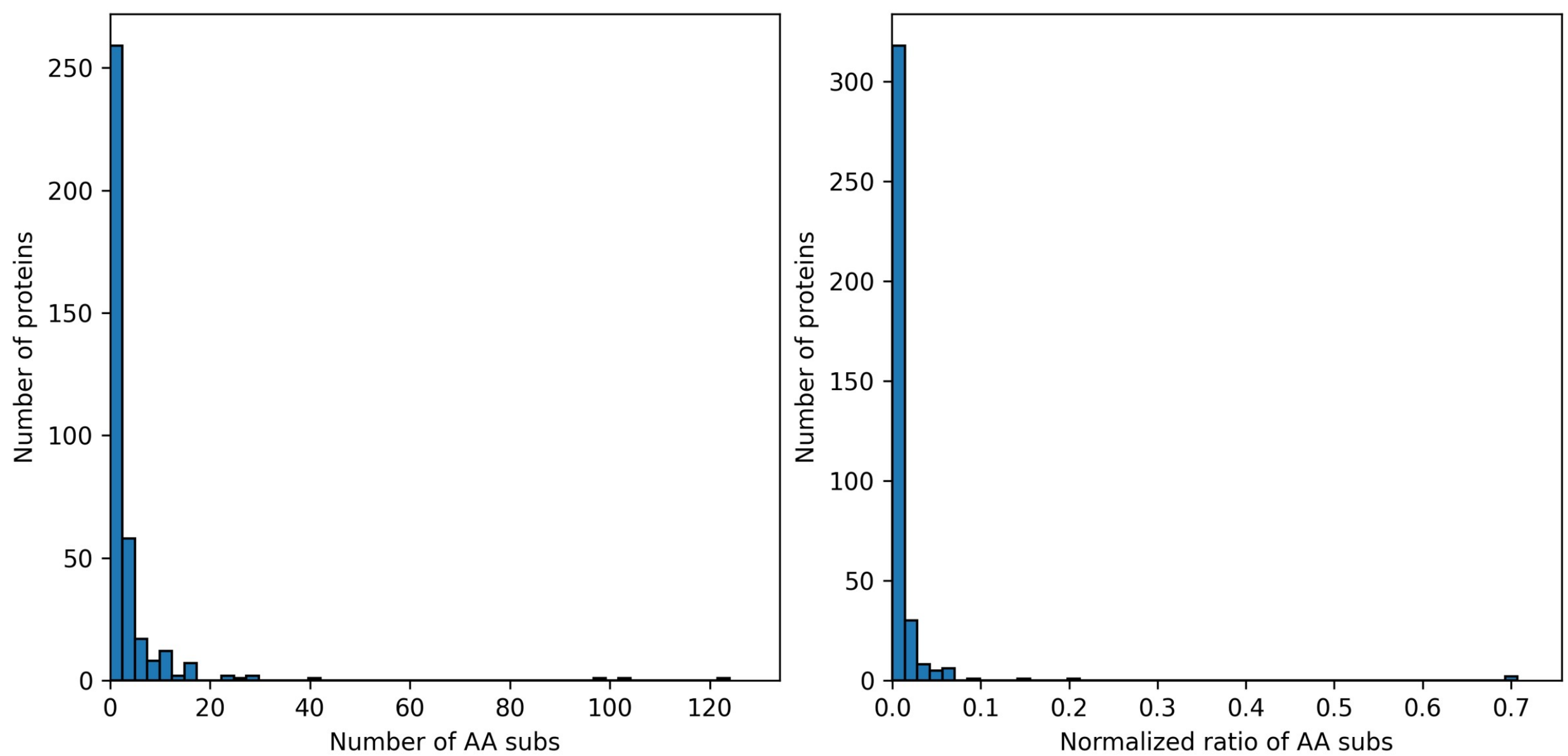

Figure S10: The histogram of the number of identified AA substitutions in proteins. Left: before normalization by the protein length. Right: after normalization by the protein length.

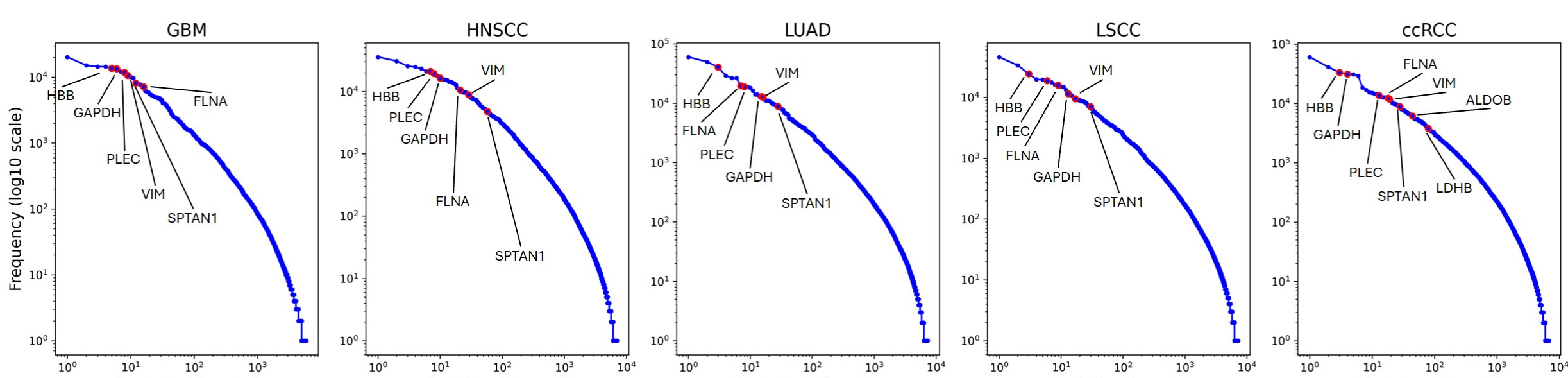

Figure S11: The abundance of the proteins that have multiple significantly regulated AA substitutions

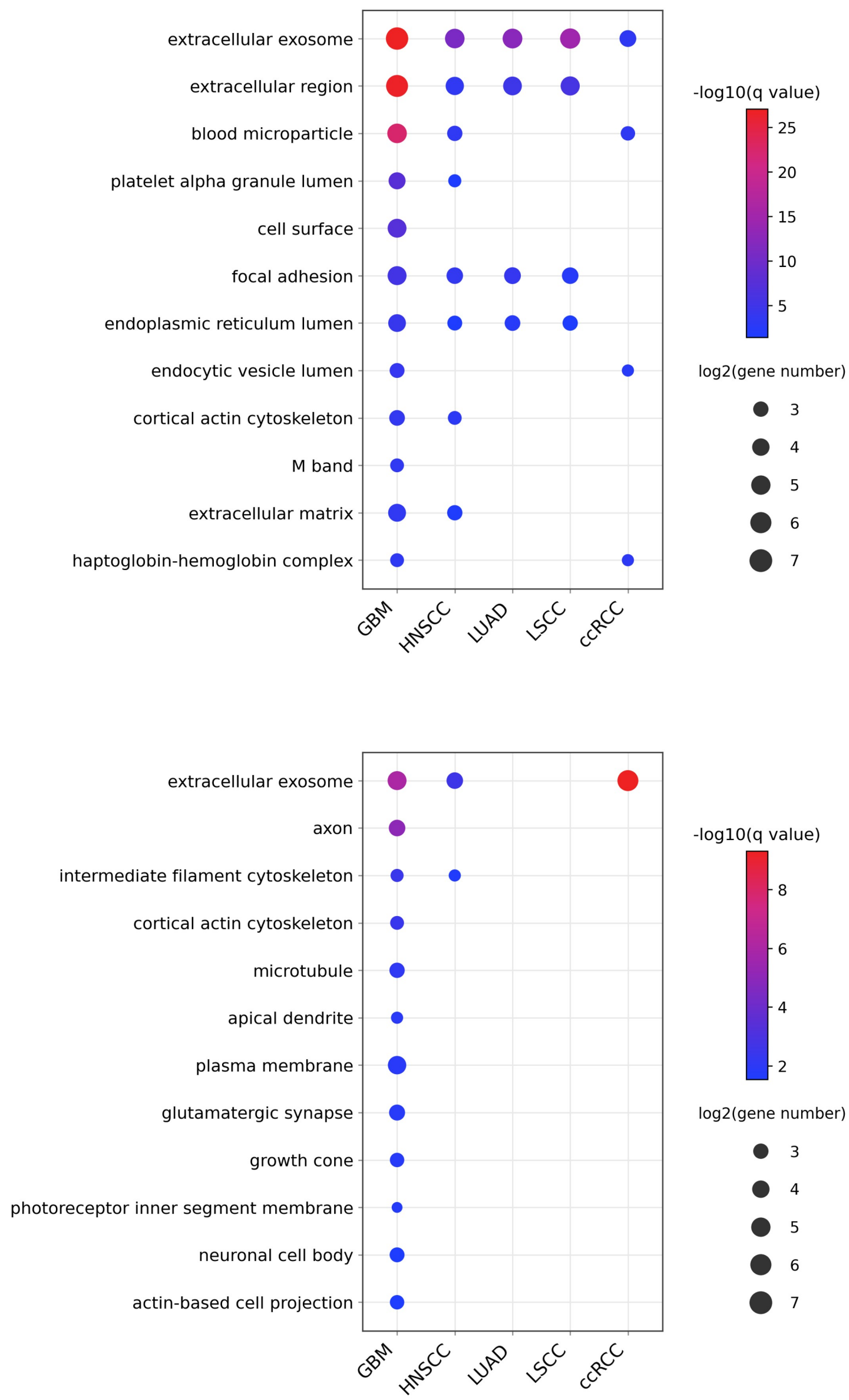

Figure S12: The GO cellular component enrichment results based on the significantly regulated UPSPs. Enrichment was computed against the proteins identified in the corresponding cohort as background, with the foreground defined on the limma-significant set. Top: the cellular component enrichment based on up-regulated UPSPs. Bottom: the cellular component enrichment based on down-regulated UPSPs. The size and color of the bubble represent the number of genes enriched in each cellular component and their minus base 10 logarithm of q value, respectively.

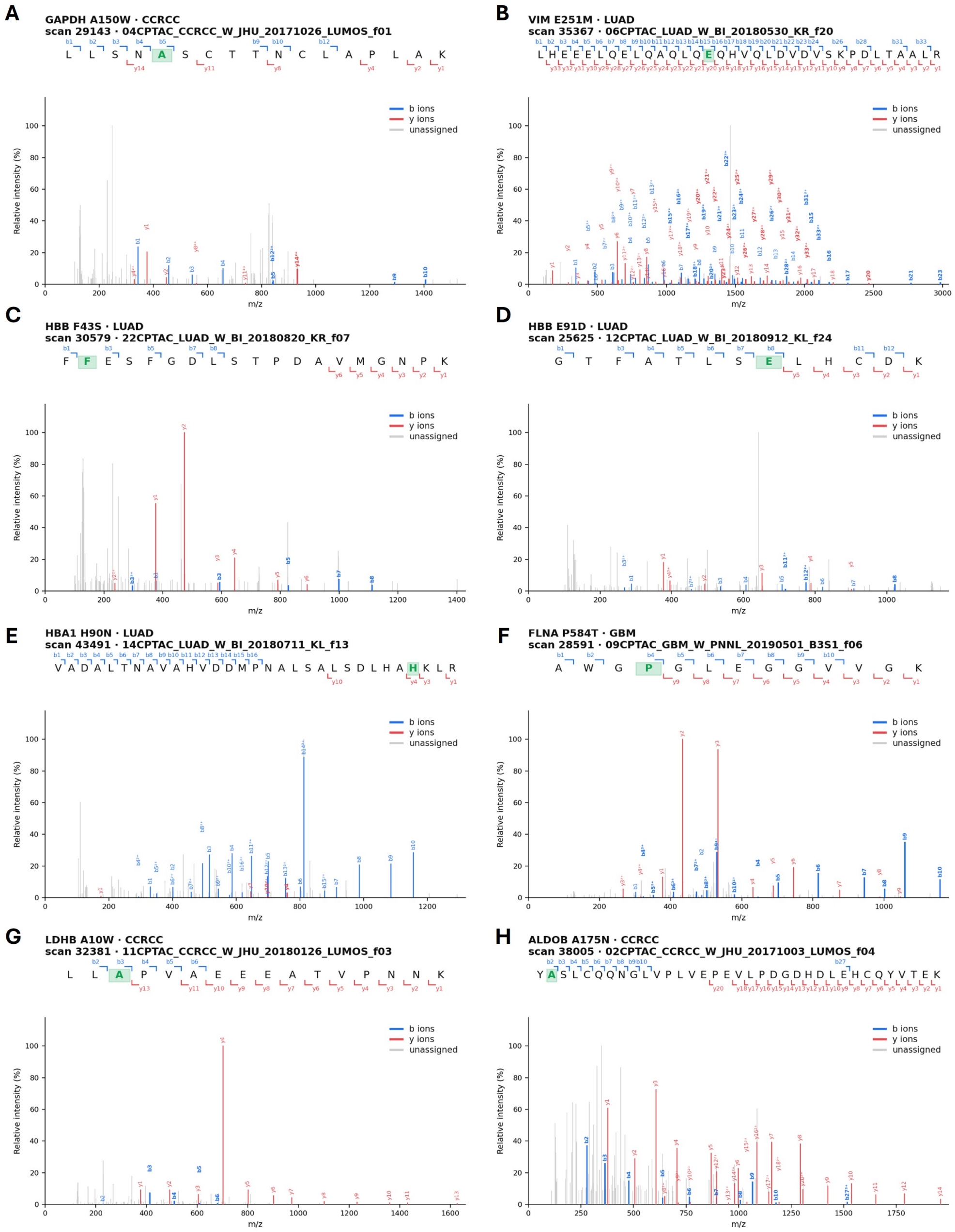

Figure S13: Manually annotated MS/MS spectra supporting eight representative AA substitutions. Each panel shows the annotated spectrum of the highest-scoring PSM for the indicated substitution, with matched b- and y-ions labelled and the sequence ladder shown above. Ions that span the substituted residue and therefore localize the mass shift are highlighted by green. The b/y ion coverage ranges from 61% to 100%; each substitution is spanned by at least two site-determining fragment ions. Per-spectrum metrics are as follows (protein, substitution, cohort, charge, precursor mass error, b/y ion coverage): GAPDH A150W, ccRCC, 3+, -6.6 ppm, 75%; VIM E251M, LUAD, 3+, +3.0 ppm, 100%; HBB F43S, LUAD, 3+, -6.6 ppm, 61%; HBB E91D, LUAD, 3+, +0.7 ppm, 92%; HBA1 H90N, LUAD, 6+, -2.3 ppm, 67%; FLNA P584T, GBM, 3+, -1.9 ppm, 92%; LDHB A10W, ccRCC, 2+, -8.1 ppm, 93%; ALDOB A175N, ccRCC, 3+, -2.0 ppm, 82%.

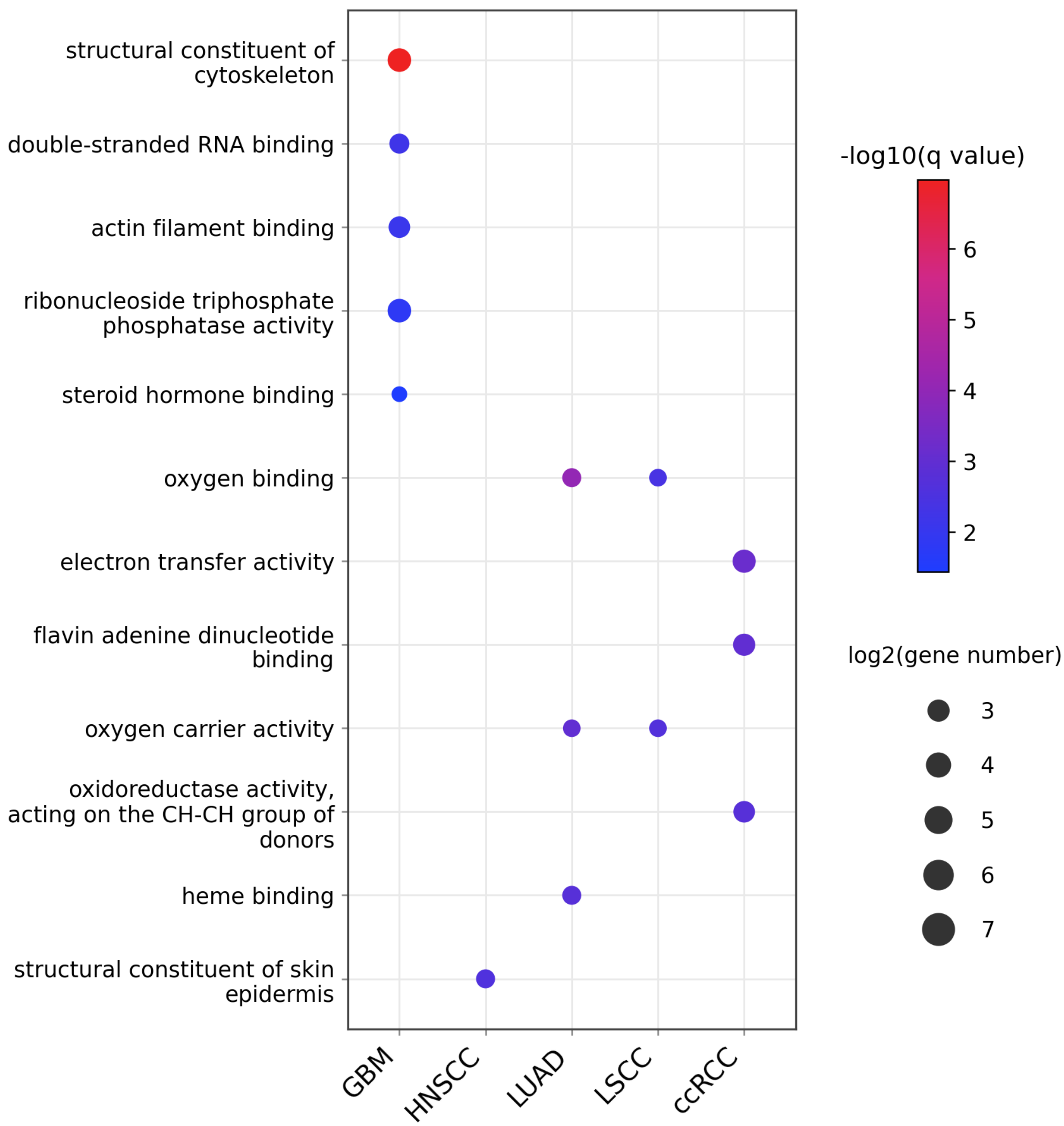

Figure S14: The GO molecular function enrichment results based on the down-regulated UPSPs. Enrichment was computed against the proteins identified in the corresponding cohort as background, with the foreground defined on the limma-significant set. The size and color of the bubble represent the number of genes enriched in each molecular function and their minus base 10 logarithm of q value, respectively.

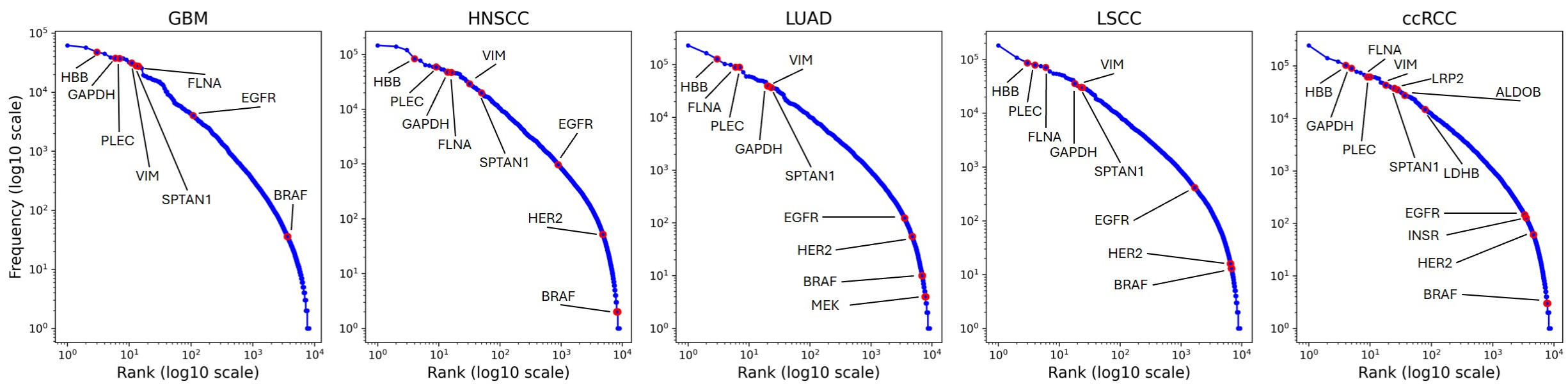

Figure S15: The abundance of the proteins that have multiple AA substitutions and the cancer drug target proteins from the PIPI-C's identification results.

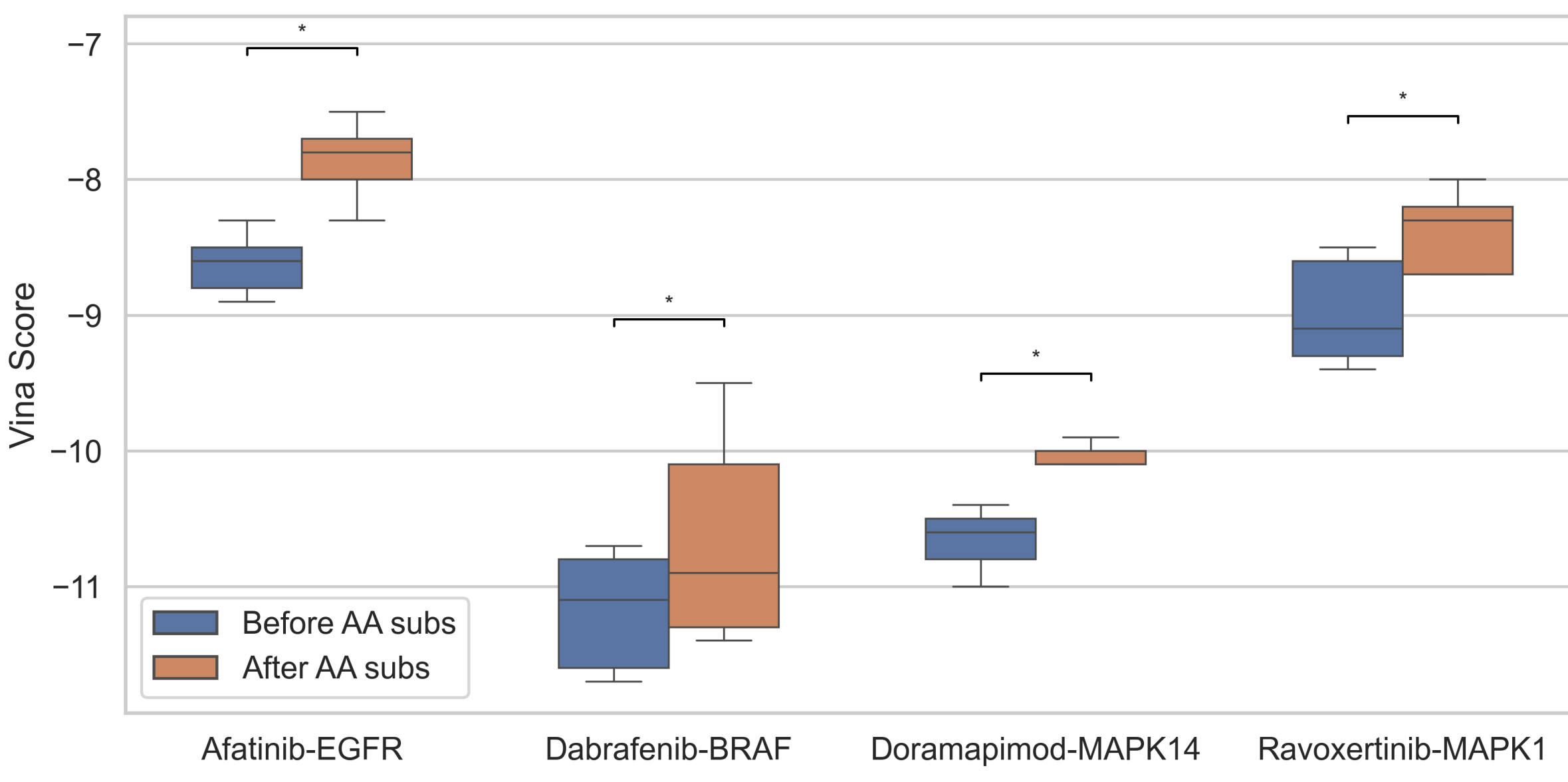

Figure S16: The change of Vina score change before and after the AA substitutions of the four drug-target pairs. The observed changes are presented as hypothesis-generating rather than as evidence of altered binding affinity. T-test p-values are shown, $\star$: p-value < 0.05.

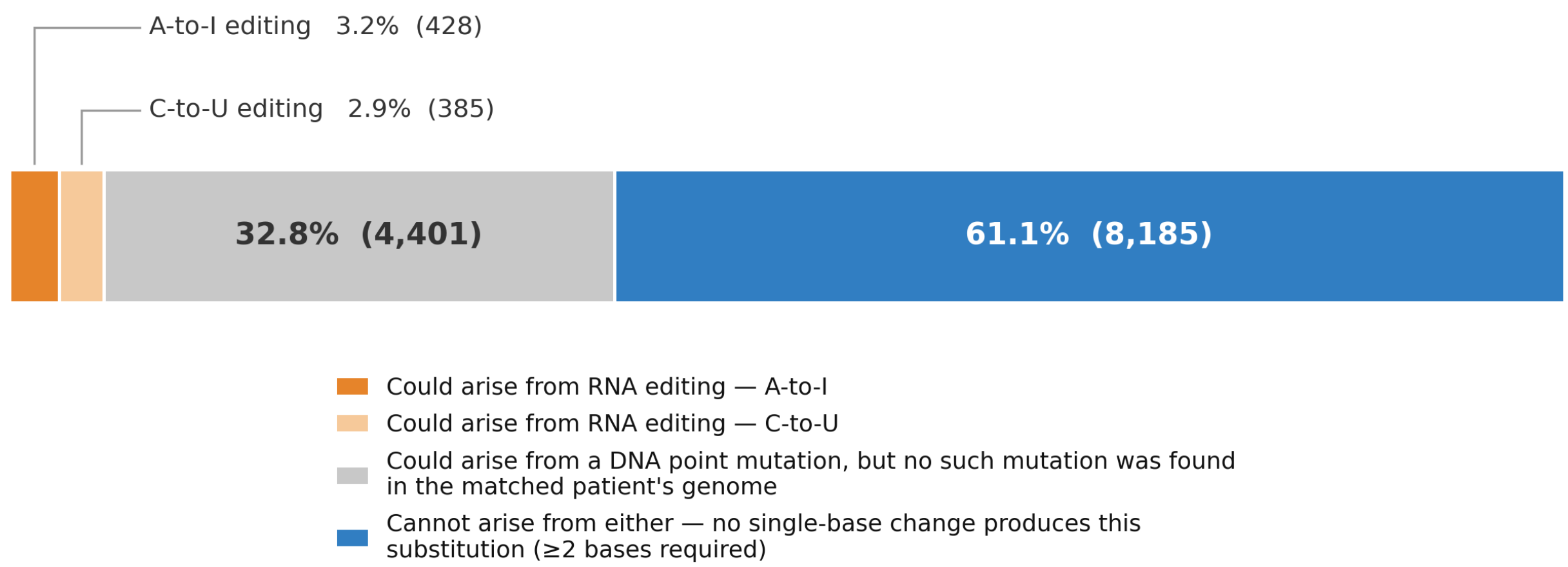

Figure S17: Most identified AA substitutions cannot be explained by matched somatic mutations or by single canonical RNA-editing events. Each of the 13,399 identified AA substitutions (five CPTAC cohorts) was cross-referenced against the matched whole-exome somatic mutation calls for the same patient and classified by whether it could be produced by a single-nucleotide change. None (0 of 13,399, 0.0%) matched a somatic missense mutation at the same position and AA change in the matched patient, and this class therefore has zero width in the bar. The four categories shown are mutually exclusive and sum to 100%. Substitutions consistent with A-to-I (codon A>G route; 3.2%, n = 428; deep orange) or C-to-U (codon C>T route; 2.9%, n = 385; light orange) editing are upper bounds: codon-level consistency indicates that such an edit could produce the observed change, not that editing occurred, and no matched RNA-seq was found. A-to-I and C-to-U were considered because they are the best-characterized recoding RNA-editing mechanisms in human coding transcripts. Grey (32.8%, n = 4,401) denotes substitutions reachable by a single DNA base change for which no corresponding mutation was found in the matched genome. Blue (61.1%, n = 8,185) denotes substitutions that require at least two base changes and therefore cannot arise from either a single somatic point mutation or a single RNA-editing event. Per-substitution results are in Supplementary File 13.

7 Supplementary Note 7: Detailed per-protein AA-substitution descriptions

This note collects the residue-by-residue substitution profiles, structural-interface analyses, and supporting literature for the proteins summarized in the main-text sections on extracellular exosome, actin filament binding, and glycolysis/gluconeogenesis proteins. As stated in the main text and Methods, each AlphaFold3 model incorporates all identified substitutions to visualize cumulative structural effects and does not represent a dominant proteoform, as individual substitutions occur at low stoichiometry across a heterogeneous population of protein molecules.

7.1 Extracellular exosome proteins

GAPDH. For GAPDH, we mapped ten AA substitutions in GBM, one in LUAD, four in LSCC, and two in ccRCC (Figure 3C, Supplementary File 2). A functional interaction region spanning residues 2 to 148 in GAPDH mediates binding with tryptophanyl-tRNA synthetase 1 (WARS1), a critical enzyme regulating aminoacylation during protein translation.[18] Within this region, we identified four upregulated AA substitutions in GBM (sites 100, 101, 106, 119) and two upregulated substitutions in LSCC (sites 79, 110). These substitutions are predicted to disrupt the GAPDH-WARS1 interaction and impair downstream aminoacylation activity, altering global protein synthesis in tumor cells.

VIM. For VIM, substitutions at 23 sites were upregulated (Figure 3D), while two distinct substitution events were detected at site 251: E251M (16-fold change) and E251F (5.54-fold change) (Supplementary File 2). The intermediate filament (IF) rod domain, spanning residues 103 to 411, constitutes 66% of the full VIM sequence and harbors 19 of the identified substitution sites; this domain forms the core structural scaffold of VIM and is essential for cytoskeletal microtubule assembly and cellular structural integrity.[19] Within this rod domain, a coiled-coil region spans residues 154 to 245. To evaluate the structural impact of AA substitutions on this region, we

utilized AlphaFold3 to predict the structure of the modified sequence spanning residues 144 to 251 (carrying the E251M substitution, given its higher fold change) and compared it to the corresponding wild-type X-ray diffraction structure[20] (Figure 3E). Six upregulated AA substitutions fall within this sequence range, and the cumulative model illustrates potential structural effects if these substitutions co-occurred; because they are more likely to occur individually at low stoichiometry, this should be read as illustrative rather than as a dominant proteoform.

HBB (regulation profile). Notably, the two lung cancer cohorts (LUAD and LSCC) exhibit significant downregulation of AA substitutions specifically in HBB. In LUAD, 11 of 25 significantly downregulated UPSPs map to HBB, while in LSCC, 17 of 39 downregulated UPSPs are derived from HBB. Additionally, seven UPSPs from hemoglobin subunit alpha 1 (HBA1) are significantly downregulated in LSCC; neither HBB nor HBA1 shows similar downregulation in the other three cancer types. These two proteins are the primary subunits of hemoglobin, responsible for pulmonary oxygen transport to peripheral tissues, and their coordinated downregulation of AA substitutions in lung cancers suggests a disease-specific functional signature. This finding may provide novel proteomic-based strategies for lung cancer diagnosis and targeted therapy. HBB carries one of the highest densities of identified AA substitutions, all of the significantly regulated ones are downregulated and exclusive to the two lung cancer cohorts. All detected HBB substitutions localize to its globin domain (residues 3 to 147) (Figure 3F), with downregulated substitutions at 15 sites in LSCC and 11 sites in LUAD. Among these, sites 44 and 56 correspond to known DNA mutation-driven AA substitutions (E44K and M56I, respectively) documented in previous studies[21] (Supplementary File 2). Two substitution sites in LSCC carry two distinct substitution events: site 69 harbors L69G (0.28-fold) and L69Q (0.30-fold), while site 118 carries H118N (0.20-fold) and H118E (0.25-fold). Several downregulated substitutions are shared between LUAD and LSCC, including A11Q, G17Q, and F43S, which may represent conserved molecular mechanisms underlying lung cancer pathogenesis.

HBB F43S (literature validation). We identified two well-validated AA substitution sites in HBB through a comprehensive literature review. Manually annotated MS/MS spectra for these and six other representative substitutions, showing the fragment ions that localize each mass shift,

are provided in Figure S13. The F43S substitution has been shown via peptide fingerprint analysis to alter the heme pocket configuration, leading to severe hemolytic anemia.[22,23] The F43 residue in HBB is evolutionarily conserved and plays a crucial role in maintaining hemoglobin structural integrity via its hydrophobic properties. Substitution with a polar serine residue induces significant conformational changes, destabilizing the hemoglobin quaternary structure. This phenylalanine residue is strategically positioned adjacent to the heme group's porphyrin ring, facilitating optimal planar orientation and strong van der Waals interactions critical for molecular stability.[23] The introduction of a polar serine residue disrupts these favorable van der Waals interactions and weakens two key intra-chain hydrogen bonds (F43–G47 and F43–T39), further destabilizing the protein architecture. Given the porphyrin ring's essential role in oxygen binding,[24] this substitution may impair oxygen transport efficiency, with critical implications for the hypoxic tumor microenvironment in lung cancer patients.

HBB E91D (literature validation). Another well-characterized AA substitution in HBB is E91D, which has been shown to enhance oxygen-carrying efficiency via liquid chromatography (LC) profiling.[25] Although residue 91 is surface-exposed and does not directly interact with the heme pocket, it is located proximal to the heme-bound H93 and adjacent to two hydrophobic residues (L89, L92) within the heme-binding pocket. Unlike the reduced oxygen affinity associated with E91L and E91G substitutions, E91D confers increased oxygen affinity. Previous studies suggest that E91 elevates the pKa value of H147 in the deoxygenated state, stabilizing the protein's quaternary structure and enhancing oxygen binding affinity.[26] However, the significant downregulation of E91D observed in this study indicates reduced oxygen affinity of HBB in lung cancer tissues. Hypoxia is a well-established hallmark of multiple human malignancies, particularly lung cancers,[27,28] and this substitution may directly contribute to the formation and maintenance of the hypoxic tumor microenvironment.

7.2 Actin filament binding proteins

SPTAN1. SPTAN1 encodes the α-chain of the cytoskeletal scaffold protein spectrin and has context-dependent roles in tumor progression.[29,30] In the AA substitution analysis results of SPTAN1, we find three AA substitutions in GBM, two in HNSCC, one in LUAD, and two in LSCC. Additionally, two common AA substitutions, the I639S substitution and the Y644F substitution, are identified from GBM and HNSCC (Figure 4D, Supplementary File 2). The UPSPs with common AA substitutions show a 3.56-fold and 3.07-fold increase in abundance in GBM and HNSCC, respectively.

PLEC. Plectin (PLEC), a cytoskeletal linker present in nearly all mammalian cells,[31] carried a relatively large number of AA substitutions across four cohorts (eight upregulated in GBM, two in HNSCC, one in LUAD, four in LSCC). Three sites are recorded in OncoDB as mutation-derived (R2525C from DNA; A2836T and V3484M from DNA and mRNA)[21] (Supplementary File 4). Two binding and interaction regions in the PLEC sequence cover most of the identified AA substitutions (Figure 4E). The first region ranges from 175–400, which is an actin-binding region,[32] and in the LSCC cohort, we find an F330L substitution, which replaces an aromatic AA with a non-aromatic AA, in this region. The second region includes the sequence of 964–4574, which covers about 77% of PLEC's length. This region has been previously discovered to interact with IF proteins.[33] It includes seven AA substitutions in GBM, two AA substitutions in HNSCC, and one in LSCC, and all of the covered AA substitutions are upregulated substitutions. The interaction between PLEC and IF proteins might be influenced by the AA substitutions crowding in this region.

FLNA (substitution map). Filamin-A (FLNA) links actin filaments to membrane integrins and is involved in GBM proliferation and invasion,[34–37] so its substitutions and GBM-regulated UPSPs offer a new perspective on GBM cell migration. FLNA carried upregulated substitutions at 19 sites in GBM; in OncoDB, E798K and V1480M are recorded as DNA-derived and A366T as DNA/mRNA-derived, while the remainder are unrecorded (Supplementary File 4). Interestingly, sites 1804 and 2544 each carried two distinct substitution events in GBM (Figure 4F, Supplementary File 2), whereas each of the other three cohorts carried only a single substitution in FLNA. Some

AA substitutions are located in the regions of binding and interaction. In the actin-binding region from 2 to 274, there is a V122A substitution. In the region of interaction with furin from sequence 1490 to 1607, two AA substitutions (L1493K and H1543Y) are identified.

FLNA (Ig-domain 4–5 interface). The FLNA dimer structure has 24 immunoglobulin-like (Ig) domains, among which domain 4 is stabilized by interaction with domain 5, and the interface between domain 4 and domain 5 is governed by several hydrophobic β-sheet interactions.[38] Additionally, two AA substitutions in domains 4 and 5, P637E and V711D, respectively, have been previously reported to cause familial cardiac valvular dystrophy because these two substitutions likely change the hydrophobic core and destabilize the individual β-sheet domain formulation.[39]

FLNA P584T. One of the AA substitutions identified in FLNA has been validated by a previous study, highlighting significant biological implications. Our analysis reveals a P584T substitution in FLNA domain 4, which is a hydrophobic-to-hydrophilic substitution with a four-fold upregulation (Supplementary File 2). Although this residue does not reside at domain interfaces, its side chain orientation toward the β-sheet hydrophobic core suggests that P>T substitution may substantially perturb core packing, potentially destabilizing the β-sheet domain architecture. This conformational disruption likely disorders the FLNA-integrin interaction interface critical for β-sheet stabilization. Such structural alterations may underlie the observed enhancement of mutant FLNA's proliferative and invasive functions in GBM, since integrin activation is essential for cellular propulsion following FLNA release.[40] To evaluate these structural changes, we employ AlphaFold3[41] to model the P584T variant and compare it with the FLNA domains 3–5 X-ray diffraction structure[38] (Figure 4G), providing mechanistic insights into the substitution's functional consequences. These findings provide mechanistic insights into how specific AA substitutomes may contribute to cancer progression through structural perturbation of key cytoskeletal proteins.

7.3 Glycolysis and gluconeogenesis proteins

LDHB. We next analyzed the AA substitution profiles of LDHB and ALDOB, two AA substitution-bearing proteins enriched in the glycolysis and gluconeogenesis pathways. Among the identified AA substitutions and substitution sites in LDHB and ALDOB, some AA substitution sites have been found to have DNA mutation-caused AA substitutions.[21] Specifically, in LDHB, the DNA mutation-caused AA substitution is V136M, and in ALDOB, the DNA mutation-caused AA substitutions are P231R and A239S, according to OncoDB (Supplementary File 4). The upregulated AA substitutions identified in the four cohorts are annotated in Figure 5E, showing two AA substitutions for each cancer cohort (Supplementary File 2). The two AA substitutions of LDHB from the GBM cohort reside in the binding region of the oxidized form of nicotinamide adenine dinucleotide (NAD^+), whose sequence ranges from 31–53. LDHB reversibly interconverts lactate and NAD^+ with pyruvate and NADH.[42] The two AA substitutions could interfere with the conversion process and the glycolysis and gluconeogenesis pathways and affect energy generation in cancer cells.

ALDOB (A175N). ALDOB emerges as another protein with frequent substitutions, and one of the identified AA substitution sites in ALDOB has been validated by other research studies. Notably, we report the novel identification of an A175N substitution at a well-documented substitutional hotspot[43–45] (Figure 5F). Position 175 contains an evolutionarily conserved alanine across all aldolase isozymes, located within an α-helical region proximal to substrate-binding residues according to crystallographic studies.[46] Substituting alanine at this position alters the charge of the AA, which may disrupt the spatial configuration of neighboring residues on the β-strand of the ALDOB substrate-binding pocket responsible for ATP generation. Moreover, the A>N substitution modifies the intra-chain hydrogen bond between sites 175 and 179 while also changing the hydrophilicity of residue 175.[47] These alterations potentially impact molecular stability and consequently influence the glycolytic and gluconeogenic pathways, ultimately impairing energy production in cancer cells. All identified AA substitutions in ALDOB are exclusively found within the renal cancer cohort and exhibit a consistent downregulation (Supplementary File 2). This finding aligns with previous research, which links the downregulation of ALDOB to pathological characteristics observed in

ccRCC patients.[48]

ALDOB (structure). Recently, the experimental structure of human kidney ALDOB was proposed (PDB: 8D44). To exhibit the influence of the AA substitutions in this study on protein structure, we present a structural comparison between the experimental structure and the AlphaFold3-predicted structure with cumulative AA substitutions (Figure 5G). Molecular analysis of these newly discovered AA substitutions and functional analysis of the AA substitution-modified ALDOB remain to be performed. These findings provide novel insights into how AA substitutomes may contribute to metabolic reprogramming in cancer. Further functional characterization of these substitutions will be essential to fully elucidate their roles in cancer metabolism.

8 Supplementary Note 8: Candidate AA substitutions in MHC/HLA proteins

Cancer immune escape—whereby tumor cells avoid immune recognition, in part through altered antigen presentation by MHC molecules—is a hallmark of tumor progression and a key target for immunotherapy.[49,50] AA substitutions in tumor-derived proteins have been proposed to affect the antigen presentation pathway. These substitutions may alter the peptide-MHC interaction by modifying critical anchor residues that are vital for stable binding within the MHC groove. Even a single substitution at these locations can substantially decrease the binding affinity of a peptide, thereby hindering its stable presentation on the tumor cell surface and rendering it undetectable to T cells.[51] Computational tools such as NetMHCpan have become essential for predicting peptide-MHC binding affinities and for identifying neoantigens—mutated peptides that are unique to tumors.[52]

For those significantly AA-substitution-modified proteins from CPTAC cohorts, we find two proteins, PLEC and LDHB, that can bind with MHC proteins. Therefore, we compare the MHC binding affinities of the modified sequences with their corresponding wild-type sequences (Supplementary File 6). The outputs from NetMHCpan[52] indicate a marginally reduced number of MHC binders (from 90 to 86 for PLEC and from 8 to 7 for LDHB). These are computational predictions on candidate substitutions and were not experimentally validated; given the small magnitude of the change, we do not interpret them as evidence of altered antigen presentation.

To further investigate the impact of AA substitutions on MHC proteins, we collected and analyzed the MHC protein-enriched datasets. The datasets were collected from ProteomeXchange with the identifiers PXD003790[53] and PXD000394.[54] These immunopeptidome datasets were not derived from immune-escaped tumors; therefore, the MHC-protein substitutions identified here should be interpreted as candidates. The search results are available on Zenodo, with record number 17669609. Because the HLA loci are the most polymorphic in the human genome, we re-examined every substitution at the residue level against both the source cell lines' own germline alleles and

the complete IPD-IMGT/HLA catalogue. Three substitutions corresponded exactly to the source cell line's own allele and have been removed from the following analysis (Supplementary File 12). For the remaining 42 substitutions, they are displayed in **Figure S18A–C** as the cumulative AA substitution maps for HLA-A, HLA-B, and HLA-C, respectively (Supplementary File 7). Furthermore, **Figure S18D–F** presents structural comparisons between the original protein structures and the AlphaFold3 predicted models for these MHC proteins, emphasizing how the AA substitutions affect interactions with presented peptides.

HLA molecules present tumor-derived antigens to cytotoxic T cells, binding peptides in a groove whose anchor pockets are formed by three α domains.[55] Substitutions within these α domains can alter their physicochemical properties and, in principle, the peptide repertoire, potentially impairing antigen presentation.[56,57] In this study, the majority of identified AA substitutions in MHC proteins are situated within the α domains. Specifically, HLA-A contains 7 AA substitutions within its α domains (**Figure S18A**), HLA-B has 13 substitutions (**Figure S18B**), and HLA-C features 12 AA substitutions (**Figure S18C**). AA substitutions falling within the α domains could in principle alter the physicochemical properties of the anchor pockets, although we did not test whether the peptide repertoire is affected.

We next examined where the retained substitutions lie relative to the peptide-binding groove, using the 34 peptide-contacting positions that define the MHC binding pocket. We found 3 of the 42 retained substitutions fall on a pocket-contacting residue (HLA-B site 138, HLA-C site 86, and HLA-C site 182). We further predicted binders for the wild-type and substituted HLA sequences with NetMHCpan (Supplementary File 8). For HLA-A, its predicted binders remained unchanged after substitution. The three pocket-contacting substitutions in HLA-B and HLA-C mark candidate sites for altered peptide presentation; NetMHCpan does not return calibrated ranks for these substituted sequences, so we report them as candidates rather than a quantified affinity change.

Functional characterization of these substitutions remains to be performed, and the present data do not establish that any of them could significantly affect antigen presentation. What this analysis

does establish is a boundary condition for AA substitutomics: at hyperpolymorphic loci, apparent substitutions cannot be interpreted without subject-specific databases.

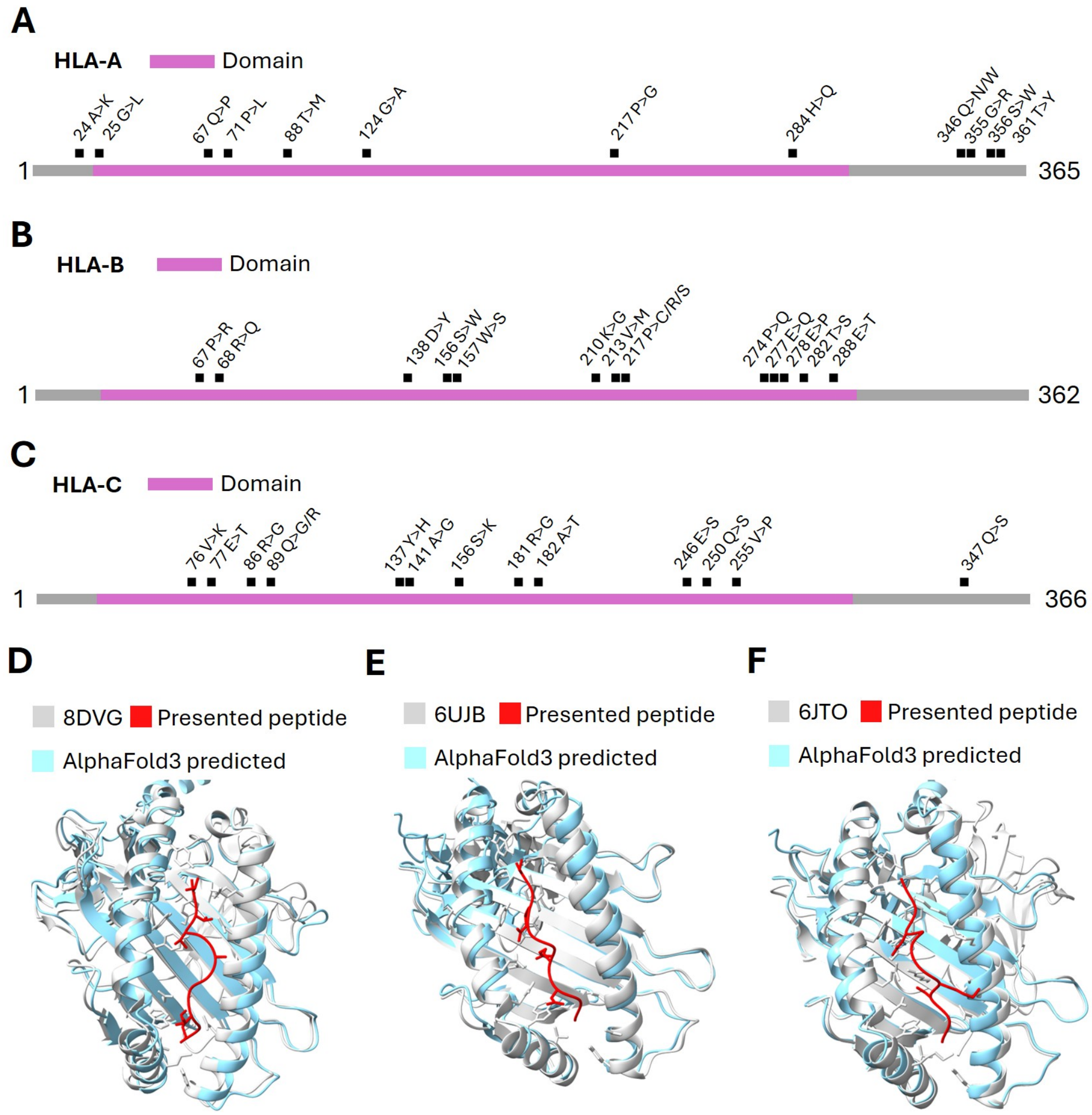

Figure S18: AA substitutomics highlights candidate substitutions in MHC/HLA proteins.
(A) Cumulative AA substitution map of HLA-A (Supplementary File 7). Pink segment: Alpha domains in the MHC class I protein.
(B) Cumulative AA substitution map of HLA-B (Supplementary File 7). Pink segment: Alpha domains in the MHC class I protein.
(C) Cumulative AA substitution map of HLA-C (Supplementary File 7). Pink segment: Alpha domains in the MHC class I protein.
(D) Structure comparison between the X-ray diffraction structure of human HLA-A (PDB: 8DVG) and the structure prediction of HLA-A with cumulative AA substitutions. Gray: the structure of 8DVG. Light blue: AlphaFold3-predicted structure with cumulative AA substitutions, which does not correspond to a predominant proteoform. Red: presented peptide (VVVGAGGVGK).
(E) Structure comparison between the electron microscopy structure of human HLA-B (PDB: 6UJB) and the structure prediction of HLA-B with cumulative AA substitutions. Gray: the structure of 6UJB. Light blue: AlphaFold3-predicted structure with cumulative AA substitutions, which does not correspond to a predominant proteoform. Red: presented peptide (SPNGTIRNIL).
(F) Structure comparison between the X-ray diffraction structure of human HLA-C (PDB: 6JTO) and the structure prediction of HLA-C with cumulative AA substitutions. Gray: the structure of 6JTO. Light blue: AlphaFold3-predicted structure with cumulative AA substitutions, which does not correspond to a predominant proteoform. Red: presented peptide (GADGVGKSAL).

9 Supplementary Data Files

Supplementary Data S1. Normalized ratios of each AA substitution type across the five cohorts (xlsx)

Supplementary Data S2. Quantification results for the five cohorts (xlsx)

Supplementary Data S3. AA substitution details of all proteins recorded in UniProt (xlsx)

Supplementary Data S4. AA substitution details of the eight proteins (xlsx)

Supplementary Data S5. AA substitution details of the drug-target proteins (xlsx)

Supplementary Data S6. NetMHCpan 4.2 output for PLEC and LDHB (xlsx)

Supplementary Data S7. AA substitution details of the MHC proteins (xlsx)

Supplementary Data S8. NetMHCpan 4.2 predicted binders (xlsx)

Supplementary Data S9. Benchmark of the four methods on the simulated datasets (xlsx)

Supplementary Data S10. Site- and peptidoform-level validation results (xlsx)

Supplementary Data S11. One-sample t-test, limma t-test, and mixed-effects model comparison (xlsx)

Supplementary Data S12. Cross-check of annotated AA substitution against germline HLA alleles (xlsx)

Supplementary Data S13. Cross-reference of AA substitutions against CPTAC somatic mutations (xlsx)

References

(1) Lai, S.; Dai, S.; Zhao, P.; Zhou, C.; Li, N.; Yu, W. PIPI-C: A combinatorial optimization framework for identifying post-translational modification hot-spots in mass spectrometry data. *Mol. Cell. Proteomics* **2026**, *25*, 101494.

(2) Yu, F.; Li, N.; Yu, W. PIPI: PTM-invariant peptide identification using coding method. *J. Proteome Res.* **2016**, *15*, 4423–4435.

(3) Lai, S.; Zhao, P.; Zhou, C.; Li, N.; Yu, W. PIPI2: Sensitive tag-based database search to identify peptides with multiple post-translational modifications. *J. Proteome Res.* **2024**, *23*, 1960–1969.

(4) Ferragina, P.; Manzini, G. In *Proceedings 41st Annual Symposium on Foundations of Computer Science*, 2000, pp 390–398.

(5) Gurobi Optimization, LLC Gurobi Optimizer Reference Manual, 2023.

(6) Zhou, C.; Dai, S.; Lin, Y.; Lian, S.; Fan, X.; Li, N.; Yu, W. Exhaustive cross-linking search with protein feedback. *J. Proteome Res.* **2022**, *22*, 101–113.

(7) Chi, H.; Liu, C.; Yang, H.; Zeng, W. F.; Wu, L.; Zhou, W. J.; Wang, R. M.; Niu, X. N.; Ding, Y. H.; Zhang, Y., et al. Comprehensive identification of peptides in tandem mass spectra using an efficient open search engine. *Nat. Biotechnol.* **2018**, *36*, 1059–1061.

(8) Na, S.; Kim, J.; Paek, E. MODplus: Robust and unrestrictive identification of post-translational modifications using mass spectrometry. *Anal. Chem.* **2019**, *91*, 11324–11333.

(9) Zeng, W.; Zhou, X.; Willems, S.; Ammar, C.; Wahle, M.; Bludau, I.; Voytik, E.; Strauss, M. T.; Mann, M. AlphaPeptDeep: A modular deep learning framework to predict peptide properties for proteomics. *Nat. Commun.* **2022**, *13*, 7238.

(10) Zolg, D. P.; Wilhelm, M.; Schmidt, T.; Médard, G.; Zerweck, J.; Knaute, T.; Wenschuh, H.; Reimer, U.; Schnatbaum, K.; Kuster, B., et al. ProteomeTools: Systematic characterization of 21 post-translational protein modifications by LC-MS/MS using synthetic peptides. *Mol. Cell. Proteomics* **2018**, *17*, 1850–1863.

(11) Deutsch, E. W.; Bandeira, N.; Perez Riverol, Y.; Sharma, V.; Carver, J. J.; Mendoza, L.; Kundu, D. J.; Wang, S.; Bandla, C.; Kamatchinathan, S., et al. The ProteomeXchange consortium at 10 years: 2023 update. *Nucleic Acids Res.* **2023**, *51*, D1539–D1548.

(12) Kong, A. T.; Leprevost, F. V.; Avtonomov, D. M.; Mellacheruvu, D.; Nesvizhskii, A. I. MSFragger: ultrafast and comprehensive peptide identification in mass spectrometry–based proteomics. *Nat. Methods* **2017**, *14*, 513–520.

(13) Creasy, D. M.; Cottrell, J. S. Unimod: Protein modifications for mass spectrometry. *Proteomics* **2004**, *4*, 1534–1536.

(14) Consortium, U. UniProt: A worldwide hub of protein knowledge. *Nucleic Acids Res.* **2019**, *47*, D506–D515.

(15) Chambers, M. C.; Maclean, B.; Burke, R.; Amodei, D.; Ruderman, D. L.; Neumann, S.; Gatto, L.; Fischer, B.; Pratt, B.; Egertson, J., et al. A cross-platform toolkit for mass spectrometry and proteomics. *Nat. Biotechnol.* **2012**, *30*, 918–920.

(16) Mertins, P.; Qiao, J. W.; Patel, J.; Udeshi, N. D.; Clauser, K. R.; Mani, D.; Burgess, M. W.; Gillette, M. A.; Jaffe, J. D.; Carr, S. A. Integrated proteomic analysis of post-translational modifications by serial enrichment. *Nat. Methods* **2013**, *10*, 634–637.

(17) Berg, M. D.; Zhu, Y.; Ruiz, B. Y.; Loll Krippleber, R.; Isaacson, J.; San Luis, B. J.; Genereaux, J.; Boone, C.; Villén, J.; Brown, G. W., et al. The amino acid substitution affects cellular response to mistranslation. *G3: Genes, Genomes, Genetics* **2021**, *11*, 218.

(18) Wakasugi, K.; Nakano, T.; Morishima, I. Oxidative stress-responsive intracellular regulation specific for the angiostatic form of human tryptophanyl-tRNA synthetase. *Biochemistry* **2005**, *44*, 225–232.

(19) Schaedel, L.; Lorenz, C.; Schepers, A. V.; Klumpp, S.; Köster, S. Vimentin intermediate filaments stabilize dynamic microtubules by direct interactions. *Nat. Commun.* **2021**, *12*, 3799.

(20) Aziz, A.; Hess, J. F.; Budamagunta, M. S.; Voss, J. C.; Kuzin, A. P.; Huang, Y. J.; Xiao, R.; Montelione, G. T.; Fitzgerald, P. G.; Hunt, J. F. The structure of vimentin linker 1 and

rod 1B domains characterized by site-directed spin-labeling electron paramagnetic resonance (SDSL-EPR) and X-ray crystallography. *J. Biol. Chem.* **2012**, *287*, 28349–28361.

(21) Tang, G.; Cho, M.; Wang, X. OncoDB: an interactive online database for analysis of gene expression and viral infection in cancer. *Nucleic Acids Res.* **2022**, *50*, D1334–D1339.

(22) Dacie, J.; Shinton, N.; Gaffney, P.; Carrell, R.; Lehmann, H. Haemoglobin Hammersmith (β 42 (CD1) Phe→Ser). *Nature* **1967**, *216*, 663–665.

(23) Rahbar, S.; Feagler, R.; Beutler, E. Hemoglobin Hammersmith (β42 (CDI) Phe→Ser) Associated with Severe Hemolytic Anemia. *Hemoglobin* **1981**, *5*, 97–105.

(24) Layer, G.; Reichelt, J.; Jahn, D.; Heinz, D. W. Structure and function of enzymes in heme biosynthesis. *Protein Sci.* **2010**, *19*, 1137–1161.

(25) Baklouti, F.; Giraud, Y.; Francina, A.; Richard, G.; Favre-Gilly, J.; Delaunay, J. Hemoglobin Pierre-Bénite [β90 (F6) Glu→Asp], a new high affinity variant found in a French family. *Hemoglobin* **1988**, *12*, 171–177.

(26) Wajcman, H.; Bascompte, J. A.; Labie, D.; Poyart, C.; Bohn, B. Structural and functional studies of hemoglobin Barcelona ($\alpha 2\beta 2$ 94 Asp (FG1)→His): Consequences of altering an important intrachain salt bridge involved in the alkaline Bohr effect. *J. Mol. Biol.* **1982**, *156*, 185–202.

(27) Semenza, G. L. Oxygen sensing, homeostasis, and disease. *N. Engl. J. Med.* **2011**, *365*, 537–547.

(28) Shi, Y.; Fan, S.; Wu, M.; Zuo, Z.; Li, X.; Jiang, L.; Shen, Q.; Xu, P.; Zeng, L.; Zhou, Y., et al. YTHDF1 links hypoxia adaptation and non-small cell lung cancer progression. *Nat. Commun.* **2019**, *10*, 4892.

(29) Teliska, L. H.; Rasband, M. N. Spectrins. *Curr. Biol.* **2021**, *31*, R504–R506.

(30) Ackermann, A.; Brieger, A. The role of nonerythroid spectrin αII in cancer. *J. Oncol.* **2019**, *2019*, 604.

(31) Svitkina, T. M.; Verkhovsky, A. B.; Borisy, G. G. Plectin sidearms mediate interaction of intermediate filaments with microtubules and other components of the cytoskeleton. *J. Cell Biol.* **1996**, *135*, 991–1007.

(32) Garcia Alvarez, B.; Bobkov, A.; Sonnenberg, A.; de Pereda, J. M. Structural and functional analysis of the actin binding domain of plectin suggests alternative mechanisms for binding to F-actin and integrin β4. *Structure* **2003**, *11*, 615–625.

(33) Bouameur, J. E.; Favre, B.; Fontao, L.; Lingasamy, P.; Begré, N.; Borradori, L. Interaction of plectin with keratins 5 and 14: Dependence on several plectin domains and keratin quaternary structure. *J. Invest. Dermatol.* **2014**, *134*, 2776–2783.

(34) Ketebo, A. A.; Park, C.; Kim, J.; Jun, M.; Park, S. Probing mechanobiological role of filamin A in migration and invasion of human U87 glioblastoma cells using submicron soft pillars. *Nano Converg.* **2021**, *8*, 19.

(35) Cai, L.; Li, Q.; Li, W.; Wang, C.; Tu, M.; Zhu, Z.; Su, Z.; Lu, X. Calpain suppresses cell growth and invasion of glioblastoma multiforme by producing the cleavage of filamin A. *Int. J. Clin. Oncol.* **2020**, *25*, 1055–1066.

(36) Chantaravisoot, N.; Wongkongkathep, P.; Loo, J. A.; Mischel, P. S.; Tamanoi, F. Significance of filamin A in mTORC2 function in glioblastoma. *Mol. Cancer* **2015**, *14*, 1–14.

(37) Iwamoto, D. V.; Huehn, A.; Simon, B.; Huet Calderwood, C.; Baldassarre, M.; Sindelar, C. V.; Calderwood, D. A. Structural basis of the filamin A actin-binding domain interaction with F-actin. *Nat. Struct. Mol. Biol.* **2018**, *25*, 918–927.

(38) Sethi, R.; Seppälä, J.; Tossavainen, H.; Ylilauri, M.; Ruskamo, S.; Pentikäinen, O. T.; Pentikäinen, U.; Permi, P.; Ylänne, J. A novel structural unit in the N-terminal region of filamins. *J. Biol. Chem.* **2014**, *289*, 8588–8598.

(39) Kyndt, F.; Gueffet, J. P.; Probst, V.; Jaafar, P.; Legendre, A.; Le Bouffant, F.; Toquet, C.; Roy, E.; McGregor, L.; Lynch, S. A., et al. Mutations in the gene encoding filamin A as a cause for familial cardiac valvular dystrophy. *Circulation* **2007**, *115*, 40–49.

(40) Savoy, R. M.; Ghosh, P. M. The dual role of filamin A in cancer: Can't live with (too much of) it, can't live without it. *Endocr. Relat. Cancer* **2013**, *20*, R341–R356.

(41) Abramson, J.; Adler, J.; Dunger, J.; Evans, R.; Green, T.; Pritzel, A.; Ronneberger, O.; Willmore, L.; Ballard, A. J.; Bambrick, J., et al. Accurate structure prediction of biomolecular interactions with AlphaFold 3. *Nature* **2024**, *630*, 1–3.

(42) Valvona, C. J.; Fillmore, H. L.; Nunn, P. B.; Pilkington, G. J. The regulation and function of lactate dehydrogenase A: Therapeutic potential in brain tumor. *Brain Pathol.* **2016**, *26*, 3–17.

(43) Cross, N.; Cox, T.; De Franchis, R.; Sebastio, G.; Dazzo, C.; Tolan, D.; Grégori, C.; Odievre, M.; Vidailhet, M.; Romano, V., et al. Molecular analysis of aldolase B genes in hereditary fructose intolerance. *Lancet* **1990**, *335*, 306–309.

(44) Esposito, G.; Imperato, M. R.; Ieno, L.; Sorvillo, R.; Benigno, V.; Parenti, G.; Parini, R.; Vitagliano, L.; Zagari, A.; Salvatore, F. Hereditary fructose intolerance: Functional study of two novel ALDOB natural variants and characterization of a partial gene deletion. *Hum. Mutat.* **2010**, *31*, 1294–1303.

(45) Lau, J.; Tolan, D. Screening for hereditary fructose intolerance mutations by reverse dot-blot. *Mol. Cell. Probes* **1999**, *13*, 35–40.

(46) Sygusch, J.; Beaudry, D.; Allaire, M. Molecular architecture of rabbit skeletal muscle aldolase at 2.7-A resolution. *Proc. Natl. Acad. Sci. U. S. A.* **1987**, *84*, 7846–7850.

(47) Kwon, S.; Safer, J.; Nguyen, D. T.; Hoksza, D.; May, P.; Arbesfeld, J. A.; Rubin, A. F.; Campbell, A. J.; Burgin, A.; Iqbal, S. Genomics 2 Proteins portal: A resource and discovery tool for linking genetic screening outputs to protein sequences and structures. *Nat. Methods* **2024**, *21*, 1–11.

(48) Shao, Y.; Wu, B.; Yang, Z.; Liu, Z.; Ma, Y.; Huang, H.; Liu, Y.; Wang, Z.; Hu, W.; Wang, Y., et al. ALDOB represents a potential prognostic biomarker for patients with clear cell renal cell carcinoma. *Transl. Androl. Urol.* **2023**, *12*, 549.

(49) Estephan, H.; Tailor, A.; Parker, R.; Kreamer, M.; Papandreou, I.; Campo, L.; Easton, A.; Moon, E. J.; Denko, N. C.; Ternette, N., et al. Hypoxia promotes tumor immune evasion by suppressing MHC-I expression and antigen presentation. *EMBO J.* **2025**, *44*, 903–922.

(50) Marty, R.; Kaabinejadian, S.; Rossell, D.; Slifker, M. J.; van de Haar, J.; Engin, H. B.; de Prisco, N.; Ideker, T.; Hildebrand, W. H.; Font-Burgada, J., et al. MHC-I genotype restricts the oncogenic mutational landscape. *Cell* **2017**, *171*, 1272–1283.

(51) Cai, Y.; Li, D.; Lv, D.; Yu, J.; Ma, Y.; Jiang, T.; Ding, N.; Liu, Z.; Li, Y.; Xu, J. MHC-I-presented non-canonical antigens expand the cancer immunotherapy targets in acute myeloid leukemia. *Sci. Data* **2024**, *11*, 831.

(52) Nilsson, J. B.; Greenbaum, J.; Peters, B.; Nielsen, M. NetMHCpan-4.2: Improved prediction of CD8+ epitopes by use of transfer learning and structural features. *Front. Immunol.* **2025**, *16*, 1616113.

(53) Shraibman, B.; Melamed Kadosh, D.; Barnea, E.; Admon, A. HLA peptides derived from tumor antigens induced by inhibition of DNA methylation for development of drug-facilitated immunotherapy. *Mol. Cell. Proteomics* **2016**, *15*, 3058–3070.

(54) Bassani Sternberg, M.; Pletscher Frankild, S.; Jensen, L. J.; Mann, M. Mass spectrometry of human leukocyte antigen class I peptidomes reveals strong effects of protein abundance and turnover on antigen presentation. *Mol. Cell. Proteomics* **2015**, *14*, 658–673.

(55) Alberts, B.; Johnson, A.; Lewis, J.; Raff, M.; Roberts, K.; Walter, P. In *Molecular Biology of the Cell. 4th Edition*; Garland Science: 2002.

(56) Bloodworth, N.; Barbaro, N. R.; Moretti, R.; Harrison, D. G.; Meiler, J. Rosetta FlexPepDock to predict peptide-MHC binding: An approach for non-canonical amino acids. *PLoS One* **2022**, *17*, e0275759.

(57) Pidala, J.; Wang, T.; Haagenson, M.; Spellman, S. R.; Askar, M.; Battiwalla, M.; Baxter Lowe, L. A.; Bitan, M.; Fernandez Vina, M.; Gandhi, M., et al. Amino acid substitution at peptide-binding pockets of HLA class I molecules increases risk of severe acute GVHD and mortality. *Blood* **2013**, *122*, 3651–3658.
